# Laser Ultrafast Confined Alloying of Sub‐5 nm RuM (M = Cu, Rh, and Pd) Particles on Carbon Nanotubes for Hydrogen Evolution Reaction

**DOI:** 10.1002/advs.202415065

**Published:** 2025-02-21

**Authors:** Taiping Hu, Dongshi Zhang, Ningning He, Shuxian Wei, Xingyu Kang, Wei Zhang, Yunyu Cai, Yixing Ye, Pengfei Li, Changhao Liang

**Affiliations:** ^1^ Key Laboratory of Materials Physics and Anhui Key Laboratory of Nanomaterials and Nanotechnology Institute of Solid State Physics Chinese Academy of Sciences Hefei 230031 P. R. China; ^2^ Department of Materials Science and Engineering University of Science and Technology of China Hefei 230026 P. R. China; ^3^ Shanghai Key Laboratory of Materials Laser Processing and Modification School of Materials Science and Engineering Shanghai Jiao Tong University Shanghai 200240 P. R. China; ^4^ Institute for Energy Research Jiangsu University Zhenjiang 212013 P. R. China; ^5^ Lu'an Branch Anhui Institute of Innovation for Industrial Technology Lu'an 237100 P. R. China

**Keywords:** HER, Ru‐alloys, sub‐5 nm alloys, ultrafast cooling, ultrafast heating

## Abstract

Thermodynamic immiscibility is a challenge for intermetallic alloying of sub‐5 nm Ru‐based alloys, which are excellent electrochemical catalysts for water splitting. In this study, nanosecond laser ultrafast confined alloying (LUCA) is proposed to break the immiscible‐to‐miscible transition limit in the synthesis of carbon nanotubes (CNTs) supported sub‐5 nm bimetallic RuM (M = Cu, Rh, and Pd) alloy nanoparticles (NPs). The alloying of non‐noble metal Cu with varying atomic ratios of RuCu alloys is appealing owing to the low price of Cu and cost‐effective synthesis for large‐scale practical applications. Benefiting from the synergistic alloying effect and resultant H/OH binding energy alteration, the Ru_95_Cu_5_/CNTs catalysts display excellent electrocatalytic alkaline hydrogen evolution reaction (HER) activity with an overpotential of 17 mV and Tafel slope of 28.4 mV dec^−1^ at 10 mA cm^−2^, and high robustness over long‐term 5000 cyclic voltammetry cycles. The performance is much better than LUCA‐synthesized CNTs‐supported Ru_86_Rh_14_, Ru_89_Pd_11_, Ru, and Cu NPs catalysts, commercial benchmark 20% Pt/C, and other mainstream Ru‐based catalysts including wet chemistry‐synthesized RuRh particles (overpotential of 25 mV, Tafel slope of 47.5 mVdec^−1^) and RuCu/CNTs (overpotential of 39 mV) synthesized using the flash Joule heating method, indicating the great potential of LUCA for screening new classes of HER catalysts.

## Graphical abstract

In this work, the synthesis of carbon nanotubes (CNTs) supported sub‐5 nm dominant intermetallic RuCu, RuRh, and RuPd NPs are succeeded by the proposed method of nanosecond laser ultrafast confined alloying (LUCA), which is featured by its unique ultrafast heating and cooling rates and high stability for long‐term alkaline HER catalysis application.

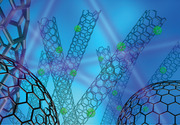

## 1 Introduction

Bimetallic alloy nanoparticles (NPs) are crucial functional nanomaterials^[^ ^1^ ^]^ that have been extensively used as catalysts for renewable energy and sustainability applications,^[^ ^1^, ^2^ ^]^ such as water splitting via the hydrogen evolution reaction (HER),^[^ ^3^ ^]^ oxygen evolution reaction (OER),^[^ ^4^ ^]^ carbon dioxide reduction reaction,^[^ ^5^ ^]^ and environmental remediation.^[^ ^6^ ^]^ Alkaline water electrolysis, currently dominated by Pt‐based nanomaterials,^[^ ^3^ ^]^ is preferred for large‐scale industrial applications. Ru‐based alloy NPs are cost‐effective alternatives to HER catalysts^[^ ^7^ ^]^ owing to the comparable hydrogen adsorption free energy (Δ*G*_H*_) of Ru with that of Pt,^[^ ^8^ ^]^ lower water‐dissociation energy barrier than that of Pt in alkaline conditions,^[^ ^7c^ ^]^ much lower cost (Ru is the cheapest Pt group metal),^[^ ^9^ ^]^ moderate hydrogen bond strength (≈ 65 kcal mol^−1^), corrosion resistance,^[^ ^10^ ^]^ and remarkably enhanced HER activities.^[^ ^11^ ^]^

**Figure 1:**
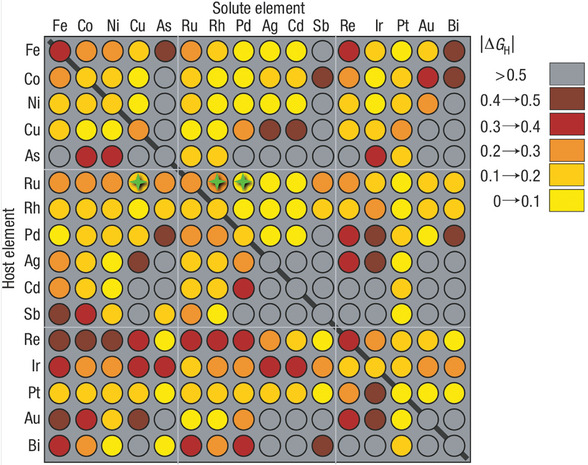
High‐throughput computational screening of ∆*G*_H_ for 256 pure metals and alloys. The diagonal plot corresponds to the ∆*G*_H_ values of pure metals. The rows indicate the pure metal substrates and the columns indicate the solutes embedded in the surface layer of the substrate. Reprinted with permission.^[^ ^12^ ^]^ Copyright 2006, Nature Publishing Group. RuCu, RuRh, and RuPd are marked by hollow green stars in the 6th row.

The Gibbs free energy of hydrogen adsorption (∆*G*_H_) is an indicator of hydrogen evolution activity, where the optimum value is 0.^[^ ^13^ ^]^ High‐throughput computational screening of ∆*G*_H_ for 256 metals/alloys (**Figure** **1**) revealed that Ru and its alloys are excellent HER electrocatalysts,^[^ ^12^ ^]^ which have been theoretically and experimentally validated.^[^ ^14^ ^]^ As marked by the green stars, the RuCu alloy has a ∆*G*_H_ value of 0.1–0.2, which lies between those of RuRh (0.2–0.3) and RuPd (0–0.1). This alloy deserves special attention because non‐noble metal Cu alloying can dramatically boost HER catalytic activity^[^ ^15^ ^]^ and reduce cost while having low toxicity and long‐term stability in alkaline electrolytes,^[^ ^14c^ ^]^ meeting the catalyst requirements for sustainable development.^[^ ^16^ ^]^ In the periodic table, Rh is located between Ru and Pd atoms. According to the “interelement fusion” principle,^[^ ^17^ ^]^ Ru–Pd alloy is a less expensive alternative to Rh,^[^ ^18^ ^]^ while the Rh–Ru alloy has the potential for multi‐catalytic catalyst development.^[^ ^19^ ^]^

Within a specific alloy‐catalyst matrix,^[^ ^20^ ^]^ small size^[^ ^21^ ^]^ and homogenous alloying^[^ ^22^ ^]^ are two desirable features for high‐performance catalyst development. However, intermetallic sub‐5 nm RuM (M = Pt, Pd, Cu) alloy NPs with high Ru:M ratios are challenging^[^ ^23^ ^]^ owing to their thermodynamic immiscibility.^[^ ^24^ ^]^ For example, in the case of Ru‐Cu, the positive formation energies,^[^ ^25^ ^]^ positive heat of formation (△H*_f_*) of +10.44 kJ mol^−1^,^[^ ^26^ ^]^ and mixing enthalpy of 7 kJ mol^−1[^ ^27^ ^]^ make it essentially immiscible.^[^ ^25^ ^]^ Despite the success in the synthesis of sub‐5 nm RuCu alloys using wet chemistry methods, the synthesis window is narrow owing to the strict screening of precursors,^[^ ^29^ ^]^ optimization of precursor ratios,^[^ ^30^ ^]^ and co‐reduction control.^[^ ^29a^ ^]^ However, intrinsic immiscibility causes Ru‐Cu more tend to form Ru@Cu core–shell and eutectic‐like structures.^[^ ^25^, ^31^ ^]^ To prevent such complex time‐consuming trial‐and‐error experiments for intermetallic Ru‐based alloying, a facile one‐step synthesis is desirable.^[^ ^14^, ^32^ ^]^ Laser irradiation in liquids^[^ ^33^ ^]^ has the potential to overcome the immiscible‐to‐miscible limit for ultrafast co‐reduction of metal precursors such as RhCl_3_ and HAuCl_4_^[^ ^34^ ^]^ and instantaneously alloy the reduced metals into free NPs. However, one‐step alloying on carbon support materials^[^ ^7a^ ^]^ that can tightly grasp NPs to inhibit their agglomeration during long‐term catalytic applications is more appealing and can also enhance interfacial conductivity and charge transport to boost catalytic performance.^[^ ^35^ ^]^

**Figure 2:**
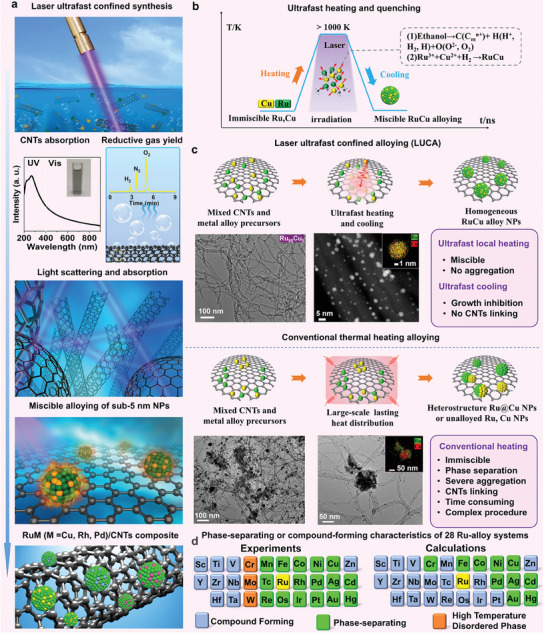
Schematic of LUCA technique for immiscible‐to‐miscible alloying of RuM NPs. a) Laser irradiation of mixed metal ions and CNTs, showing the yield of reductive H_2_ gas, light scattering/absorption of CNTs, and miscible alloying. Gas analysis of laser‐irradiated CNTs–ethanol mixture analyzed using gas chromatography. b) Ultrafast heating and cooling processes of LUCA and reduction and alloying processes of RuM‐immiscible sub‐5 nm alloy NPs. c) Schematic of LUCA versus conventional heating alloying, TEM image of synthesized CNTs‐supported Ru_95_Cu_5_ alloy NPs, and energy‐dispersive X‐ray (EDS) mapping of single alloy NP, summarizing the advantages of LUCA over conventional methods for homogeneous miscible alloying of immiscible metals. Figures S1 and S2 (Supporting Information) show the detailed structures of LUCA‐synthesized Ru_95_Cu_5_/CNTs and the conventional heating method‐synthesized Ru@Cu‐CNTs composites, respectively. The XRD spectrum shown in Figure S3 (Supporting Information) clearly indicates the phase‐separation behavior. Experimental and theoretical (ab initio calculations) phase‐separating Ru‐alloy systems. Adapted with permission.^[^ ^24^ ^]^ Copyright 2011 American Physical Society.

In this work, laser ultrafast confined alloying (LUCA) is proposed to synthesize carbon nanotubes (CNTs)‐supported intermetallic RuM (M = Cu, Rh, and Pd) alloys dominated by sub‐5 nm NPs, with a focus on miscible RuCu alloying. The concept of LUCA and its key factors are first introduced and compared with those of the conventional thermal heating method to demonstrate the merits of LUCA from the perspective of alloying capacities and states (**Figure** **2**). Afterward, we demonstrated the wide applicability of LUCA for the synthesis of multiple RuM (M = Cu, Rh, and Pd) alloys and the modulation of the size and composition of RuCu alloys. Their alkaline HER performances were studied and compared with those of commercial Pt/C, Cu/CNTs, and Ru/CNTs composites (Figure S4, Supporting Information) and other Ru‐based HER catalysts. Density functional theory (DFT) calculations were performed to elucidate the mechanism underlying the enhanced alkaline HER performances of RuCu/CNTs catalysts. The atomic ratios of the composite samples were measured by inductively coupled plasma atomic emission spectroscopy (ICP‐AES) and are listed in **Table** **1**.

**Table 1:** ICP‐AES results of LUCA‐synthesized RuCu/CNTs (synthesized at 5 mg of CNTs, 2 mg RuCl_3_, *x* mg Cu(CH_3_COO)_2_ *x* = 0.5, 1, 3, and 30 mg), RuPd/CNTs, and RuRh/CNTs.

Catalyst	Ru[wt.%]	Cu[wt.%]	Ru[at%]	Cu[at%]	Pd[wt.%]	Pd[at%]	Rh[wt.%]	Rh[at%]
Ru_97_Cu_3_/CNTs X = 0.5 mg	5.6	0.09	97.4	2.6	/	/	/	/
Ru_95_Cu_5_/CNTs X = 1 mg	4.01	0.12	95.4	4.6	/	/	/	/
Ru_89_Cu_11_/CNTs X = 3 mg	4.9	0.39	88.8	11.2	/	/	/	/
Ru_20_Cu_80_/CNTs X = 30 mg	2.6	6.64	19.8	80.2	/	/	/	/
Ru_89_Pd_11_/CNTs	4.85	/	89.1	/	0.62	10.9	/	/
Ru_86_Rh_14_/CNTs	5.03	/	86.2	/	/	/	0.82	13.8

## 2 Results and Discussion

Figure 2a shows the LUCA scheme of Ru_95_Cu_5_ alloy NPs using nanosecond (ns) laser (pulse width of 7 ns and wavelength of 355 nm) irradiation of an ethanol solvent containing CNTs adsorbents and ion precursors of immiscible Ru and Cu. CNTs are intrinsically black light absorbers,^[^ ^36^ ^]^ enabling a rapid temperature increase upon light absorption and triggering in situ photothermal heating of CNTs for miscible alloying on CNTs supports. Upon laser–CNTs interaction, the material's electrons are immediately heated, and then the energy is transferred to lattices via electron–lattice coupling, eventually reaching equilibrium. This leads to ultrafast heating of the CNTs (Figure 2b), to well above 1000 K.^[^ ^37^ ^]^ Han et al. reported that graphene oxides can be instantaneously heated to 2107 °C within 1 µs by single‐pulse ns laser irradiation.^[^ ^38^ ^]^ The heat was released into liquid, causing the surrounding ethanol molecules to decompose into gases (containing H_2_ reductive gas) and reducing the metal ions into Ru and Cu atoms. The Ru and Cu metal atoms then nucleate in the local regions of the CNTs to form adsorbed NPs. Constant stirring ensures the continuous supplementation of metal ions for ion reduction, NPs nucleation, and alloying. The alloying of immiscible metals and the prevention of phase separation and growth termination are due to ultrafast laser heating and quenching because the grain size (*d*) is linearly related to the thermal treatment time (*t*), according to $d^{n}-d_{0}^{n}=k_{0}\;exp\left(-\frac{E_{a}}{RT}\right)t$.^[^ ^39^ ^]^ In this equation, *d*_0_ and n are the initial grain size and grain growth exponent, respectively; *k*_0_ and *E*_a_ are the constant and activation energies, respectively; and *R* and *T* are the gas constant and absolute temperature, respectively. The heating and cooling rates of ns laser irradiation are on the order of 10^9^ K s^−1^,^[^ ^38^ ^]^ much faster than that of the flash carbothermic reaction, with heating/cooling rates on the order of 10^4^–10^5^ K s^−1^.^[^ ^40^ ^]^ With respect to the LUCA, the liquid environment can further accelerate the cooling rate, as evidenced by the inhibition of thermal effects during high‐quality picosecond laser manufacturing.^[^ ^41^ ^]^

LUCA‐synthesized RuCu NPs were homogeneously distributed on CNTs without distinct phase separation (Figure 2c); the majority were within the sub‐5 nm range, whereas, ultrafast flash Joule‐heating pyrolysis (heating rate of 1600 °C in 0.5 s) resulted in RuCu/CNTs composites with distinct phase separation.^[^ ^14c^ ^]^ Differently, the sequential annealing method (heating rate: 10^2^ K/h) can merely yield Pt@Ru alloy NPs,^[^ ^42^ ^]^ highlighting the importance of ultrafast heating in overcoming the immiscibility limit for intermetallic alloying. We performed thermal heat‐alloying experiments to synthesize RuCu NPs on CNTs (Figure 2c), where severe NPs aggregation and phase separation occurred, indicating the intrinsic immiscibility of Ru and Cu. The challenge lies in large‐scale ion reduction into metal atoms and their free nucleation and growth, rather than in local regions of CNTs. Large NPs aggregates also link CNTs together, in the absence of LUCA‐synthesized RuCu/CNTs, demonstrating the efficacy of LUCA for the confined miscible alloying of immiscible metals, which is challenging for conventional methods. In addition to Cu, many other metals such as Pd and Rh are also experimentally immiscible with Ru,^[^ ^24^ ^]^ as shown in Figure 2d. The mixing enthalpies of RuCu, RuPd, and RuRh are 7, 6, and 1 kJ mol^−1^, respectively, indicating that RuCu and RuPd are both intrinsically miscible, whereas RuRh with a mixing enthalpy close to 0, much easier to become miscible theoretically (Figure 2d).

**Figure 3:**
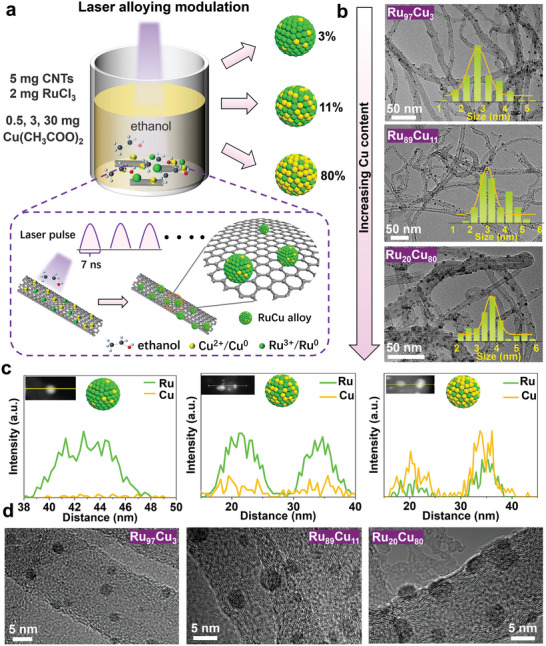
RuCu alloy composition modulation by LUCA. a) Schematic of synthesis of RuCu_x_ (x: atom percentages of 3%, 11%, 80%) alloys by changing metal ratios of the RuCl_3_ and Cu(CH_3_COO)_2_ ion precursor. b) TEM images of RuCu/CNTs and their size distributions. c) EDS line‐scanning profile (inset: high‐angle annular dark‐field (HAADF) scanning transmission electron microscopy (STEM) images showing the line scanned). d) HRTEM images of CNTs supported Ru_97_Cu_3_, Ru_89_Cu_11_, and Ru_20_Cu_80_ alloy NPs synthesized using LUCA, clearly showing that sub‐5 nm NPs constitute the majority of the products and the gradual enlargement of NPs with increasing the ratio of Cu ion precursors. The ICP‐AES results are shown in Table 1.

**Figure 4:**
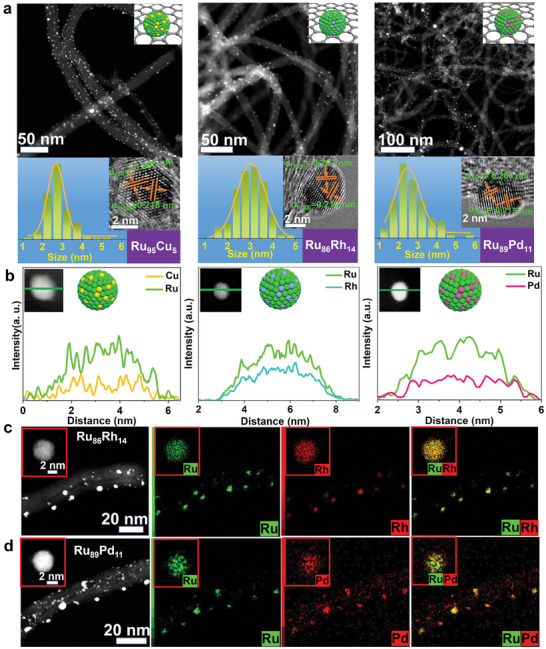
RuM/CNTs (M = Cu, Rh, and Pd) composites size/composition. a) TEM images of Ru_95_Cu_5_/CNTs, Ru_86_Rh_14_/CNTs, and Ru_89_Pd_11_/CNTs composites synthesized using LUCA, corresponding size distribution, and HRTEM images of single alloy NP. b) HAADF‐STEM images and EDS line scanning of single RuCu, RuRh, and RuPd NPs. c,d) HAADF‐STEM images and EDS mapping of Ru_86_Rh_14_/CNTs and Ru_89_Pd_11_/CNTs composites.

RuCu was adopted as a representative alloy (**Figure** **3**a,b) to demonstrate the modulation capacity of Cu to ≈ 3 at%, 11 at%, and 80 at% using LUCA while keeping the masses of the CNTs (5 mg) and RuCl_3_ ions (2 mg) constant and changing those of the Cu(CH_3_COO)_2_ ions (0.5, 1, 3, and 30 mg). The EDS line scan shown in Figure 3c indicates the trend of Cu enrichment of the RuCu alloys. The synthesized RuCu alloy particles were all within 5 nm, and no particle enlargement was evident; however, a detailed analysis clearly showed that the size of the RuCu NPs gradually increased with increasing Cu content (Figure 3b,d). This phenomenon should be attributed to the large difference in the melting points of Ru (2334 °C) and Cu (1083.4 °C), as Cu atoms are more likely to melt and enlarge the NPs. Decreasing the dimensions of an element significantly decreases its melting point, especially for metal particles. Consequently, the melting points of metallic NPs drop well below 1000 K,^[^ ^43^ ^]^ not to mention the atomic scale. To demonstrate the wide applicability of LUCA, three immiscible RuM (M = Cu, Rh, and Pd) sub‐5 nm alloy NPs were prepared. Owing to the small sizes of alloy NPs and their low mass loading, the as‐prepared alloys do not exhibit any XRD peaks except for the broad diffraction peaks arising from the CNTs (Figure S5, Supporting Information).^[^ ^44^ ^]^ Compared with RuCu NPs synthesized using wet‐chemistry methods, whose sizes and alloying states are either sensitive to precursor ion ratios^[^ ^30^ ^]^ or precursor selection,^[^ ^29b^ ^]^ LUCA is a more robust and effective synthesis technique for immiscible‐to‐miscible alloying. RuCu, RuRh, and RuPd NPs (average sizes: ≈ 3 nm), which were uniformly loaded on the CNTs (**Figure** **4**a); intermetallic alloying (Figure 4b); and the absence of severe NPs aggregation and phase separation (Figure 4c,d) indicates the wide applicability of LUCA. All the NPs had hexagonal close‐packed (hcp) crystal structures (Figure 4a).

Ultrafast (picosecond and femtosecond) laser ablation in liquids (LAL)^[^ ^45^ ^]^ excels at synthesis of sub‐10 nm particles,^[^ ^46^ ^]^ but a broad size distribution is often unavoidable owing to uncontrollable complex synthesis processes^[^ ^45^ ^]^ and two types of particle formation mechanisms.^[^ ^47^ ^]^ During the in situ synthesis of particle/CNTs nanocomposites in a CNTs matrix on carbon supports using femtosecond LAL (fs‐LAL), broad size‐distributed particles can not bond completely to the CNTs surfaces. We performed fs‐LAL of Cu in ethanol in the absence and presence of CNTs, which shows that the size of fs‐LAL synthesized Cu particles is broadly size distributed with an average size of 43.9 ± 4.67 nm and severe particle aggregation is observed (Figure S6a–c, Supporting Information). In the case of in situ fs‐LAL, high‐absorption CNTs in solution block the laser beam, leading to a reduction of the colloid productivity (Figure S6d, Supporting Information). In situ decoration of Cu particles on CNTs by fs‐LAL yields many particles >10 nm (Figure S6e,f, Supporting Information). This finding indicates that some particles are free in the solution and severely aggregate, in accordance with the states of Fe_3_O_4_/MWCNTs composites in situ synthesized by fs‐LAL in MWCNTs‐containing solution,^[^ ^48^ ^]^ highlighting the advantages of LUCA for homogeneous sub‐5 nm particle synthesis on supports. Mainstream methods for the synthesis of noble metal alkaline HER catalysts include liquid‐phase reduction, pyrolysis, hydro/solvothermal synthesis, photo/electrochemical synthesis, template‐assisted synthesis, and atomic layer deposition.^[^ ^49^ ^]^ The presented LUCA technique may present new opportunities for high‐performance alkaline HER catalyst development, which currently faces low productivity for commercialization. This can be addressable by parallel synthesis using multiple laser beams, such as high throughput multi‐beam parallel processing^[^ ^50^ ^]^ and additive manufacturing.^[^ ^51^ ^]^

**Figure 5:**
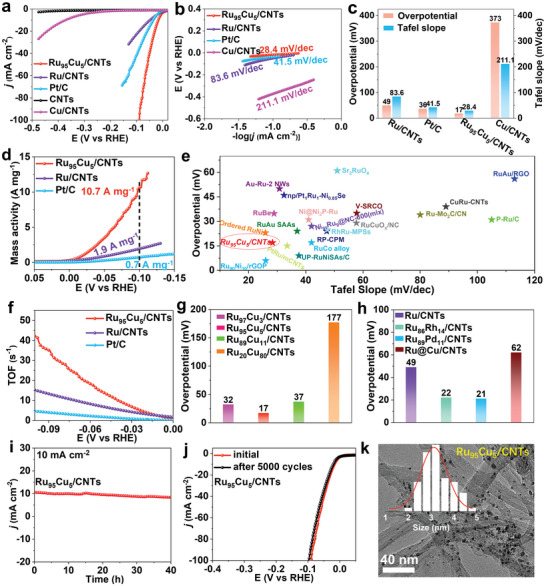
HER evaluation of LUCA‐synthesized metal/CNTs catalysts in Ar‐saturated 1.0 m KOH and performance comparison. a) LSV curves of Ru_95_Cu_5_/CNTs, Ru/CNTs, Pt/C, CNTs, and Cu/CNTs. b) Tafel slopes. c) Summarized overpotentials and Tafel slopes. d) Mass activity of Ru_95_Cu_5_/CNTs, Ru/CNTs, and Pt/C catalysts. e) Overpotentials and Tafel slopes of Ru_95_Cu_5_/CNTs and other previously reported Ru‐based HER catalysts. f) TOFs of the catalysts. g,h) Overpotentials of other LUCA‐synthesized RuCu/CNTs, Ru_86_Rh_14_/CNTs, Ru_89_Pd_11_/CNTs catalysts, and Ru@Cu/CNTs synthesized using the conventional heating synthesis method. i,j) CV tests and long‐term *i–t* of Ru_95_Cu_5_/CNTs catalyst for stability evaluation. k) TEM image and size distribution of Ru_95_Cu_5_/CNTs catalyst after stability test.

The alkaline HER performances of the CNTs, 20% Pt/C, LUCA‐synthesized Ru_95_Cu_5_/CNTs, Cu/CNTs, and Ru/CNTs in an Ar‐saturated 1.0 m KOH solution were evaluated for catalytic applications. **Figure** **5**a shows the specific activities of the catalysts normalized by their geometric areas, with the corresponding Tafel slopes and overpotentials, which are summarized in Figure 5b,c, respectively. The CNTs were ineffective HER catalysts. Compositing CNTs with Cu NPs, Ru NPs, and RuCu alloy NPs greatly enhanced the catalytic activity in the order of RuCu > Ru > Cu NPs, indicating the efficacy (synergistic effect) of RuCu alloying for HER enhancement. The Ru_95_Cu_5_/CNTs catalyst has the lowest overpotential of 17 mV and Tafel slope of 28.4 mV dec^−1^ at a current density of 10 mA cm^−2^. Although the Ru loading (11.2 µg cm^−2^) in the Ru_95_Cu_5_/CNTs catalyst is much lower than that of the commercial 20% Pt/C catalyst (56.0 µg cm^−2^), the overpotentials and Tafel slopes are much lower than those of Pt/C in 1.0 m KOH (36 mV and 41.5 mV dec^−1^). A lower Tafel slope indicates that the current density increases more rapidly as the catalyst potential decreases. The Ru_95_Cu_5_/CNTs catalyst also has a higher mass activity of 10.7 A mg^−1^ at the potential of −0.1 V versus RHE than those of the 20% Pt/C (0.7 A mg^−1^) and Ru/CNTs (1.9 A mg^−1^) catalysts (Figure 5d). Quantification of the catalyst mass was confirmed by the complete dissolution (appearance of CNTs) of the metals in aqua regia, as shown in Figure S7, Supporting Information.

Figure 5e and Table S1 (Supporting Information) summarize the overpotentials of Ru_95_Cu_5_/CNTs and other catalysts, clearly showing that Ru_95_Cu_5_/CNTs is among the best Ru‐based catalysts ever reported, such as Au–Ru nanowires (NWs),^[^ ^52^ ^]^ Ni@Ni_2_P–Ru nanorods,^[^ ^53^ ^]^ ordered Ru–Ni,^[^ ^54^ ^]^ P–Ru/C,^[^ ^55^ ^]^ RuP_2_ NP–decorated carbon porous microsheets,^[^ ^56^ ^]^ np/Pt_1_Ru_1_–Ni_0.85_Se,^[^ ^57^ ^]^ Ni_91_Ru_9_@NC–600(mix),^[^ ^58^ ^]^ RuAu single–atom alloy,^[^ ^59^ ^]^ RuAu NPs on reduced graphene oxide,^[^ ^60^ ^]^ RuBe nanosheets,^[^ ^61^ ^]^ Ru–doped Mo_2_C NPs on ultrathin nitrogen‐doped carbon nanosheets,^[^ ^62^ ^]^ Sr_2_RuO_4_,^[^ ^63^ ^]^ and SrRuO_3_.^[^ ^64^ ^]^ Compared with the counterpart RuCu/CNTs (NPs: ≈ 4.3 nm in diameter) prepared by the flash Joule heating method,^[^ ^14c^ ^]^ the overpotential of the alkaline HER is much lower (17 mV vs 39 mV), which is less than half, thus demonstrating the advantage of LUCA over the flash Joule heating synthesis method for developing high‐performance Ru‐based alloy catalysts. The alkaline HER performance of the Ru_95_Cu_5_/CNTs was also similar to that of the RuPt/mCNTs catalysts.^[^ ^7a^ ^]^

Considering the much lower price of Cu(CO_2_CH_3_)_2_·xH_2_O than H_2_PtCl_6_·xH_2_O (¥ 2080 per kg vs ¥ 1195 per g), Ru_95_Cu_5_/CNTs are competitive alternatives to RuPt/CNTs catalysts for high performance, cost‐effective catalytic applications, fitting the trend in catalytic development.^[^ ^16^ ^]^ A 5at% replacement of Ru by Cu seems inconsequential, but for practical applications where cost is important, such as large‐scale production, the replacement can effectively save costs. Most importantly, this may inspire cluster^[^ ^65^ ^]^ and single‐atom/dimer catalysts^[^ ^66^ ^]^ by laser irradiation at incomparable ultrafast heating and quenching rates, which may address the metal agglomeration issue during carbon‐supported single‐atom catalyst synthesis using the thermal decomposition method.^[^ ^67^ ^]^ From these results, it is clear that the LUCA technique itself and the as‐prepared Ru‐based alloy/CNTs catalysts (facile size control and no phase separation) are, to some extent, both advantageous to conventional and burgeoning synthesis techniques and their products for HER applications.

Electrochemical impedance spectroscopy (EIS, Figure S8, Supporting Information) reveals that the Ru_95_Cu_5_/CNTs catalysts possess a lower charge‐transfer resistance than the Ru/CNTs catalyst, indicating its faster HER kinetics. The Ru_95_Cu_5_/CNTs catalyst also has a much higher electrochemical active surface area (ECSA; 59.8 m^2^ g^−1^) than those of Ru/CNTs (31.4 m^2^ g^−1^) and Pt/C (37.6 m^2^ g^−1^), as indicated by the underpotential deposition of Cu (Cu‐UPD, 420 µC cm^−2^) shown in Figures S9 and S10a (Supporting Information). This means that the Ru_95_Cu_5_/CNTs catalyst possesses the highest number of HER active sites. After ECSA normalization of the LSV curves, Ru_95_Cu_5_/CNTs were still better than Ru/CNTs and Pt/C, as shown in Figure S10b (Supporting Information). At the potential of −0.1 V versus RHE, the Ru_95_Cu_5_/CNTs catalyst exhibits a normalized current density of 17.3 mA cm^−2^, which is much higher than those of the Ru/CNTs (6.2 mA cm^−2^) and Pt/C (1.9 mA cm^−2^) catalysts. The outstanding HER performance of the Ru_95_Cu_5_/CNTs catalyst is also supported by its high turnover frequency (TOF, Figure 5f). At a potential of −0.1 V versus RHE, the Ru_95_Cu_5_/CNTs catalyst has a TOF of 42.2 H_2_ s^−1^, surpassing those of Ru/CNTs (15.2 H_2_ s^−1^) and Pt/C (4.5 H_2_ s^−1^). CV curves were measured at different scanning rates to determine the *C*_dl_ of each catalyst (Figure S11a–c, Supporting Information), as summarized in Figure S11d (Supporting Information). The *C*_dl_ value of Ru_95_Cu_5_/CNTs (10.1 mF cm^−2^) is much higher than those of Ru/CNTs (3.03 mF cm^−2^) and Pt/C (5.72 mF cm^−2^), reaffirming that Ru_95_Cu_5_/CNTs catalyst possesses the most active sites.

Reduced graphene oxide (rGO) sheets supported by Ru‐based NPs composites are also excellent HER catalysts.^[^ ^7c^ ^]^ Metal‐NPs/rGO composites are synthesizable by laser irradiation of solution containing GO‐nanosheets and metal ions precursors.^[^ ^68^ ^]^ Cost‐effective GO nanosheets were adopted for the synthesis of RuCu/rGO composites under the same synthesis condition as that of Ru_95_Cu_5_/CNTs catalysts by 30min‐LUCA. This comparison allows us to identify the influence of the type of carbon supports on the RuCu alloy states and corresponding HER performance. On rGO sheets, the loaded particles are broadly size distributed with many particles >10 nm (Figure S12a,b, Supporting Information), unlike the case of Ru_95_Cu_5_/CNTs catalyst with almost all Ru_95_Cu_5_ particles are <5 nm (Figure S12c,d, Supporting Information). Hence, the HER performance is much lower for RuCu/GO catalysts with an overpotential of 102 mV (Figure S12e, Supporting Information), highlighting the priority of CNTs over GO sheets as the supports for in situ synthesis of sub‐5 nm Ru‐based alloy particles by LUCA for HER applications. CNTs are a kind of 1D material, exposing very limited regions for light interaction, in situ metal‐ion reduction, and particle nucleation in confined regions. Their structural properties also facilitate their flexible mobility to expose different regions to prevent repetitive irradiation on the same region, thus the size of loaded particles is much smaller. GO is a kind of 2D material enabling a large‐scale reduction of metal ions into atoms during light‐matter interaction. Despite success in the synthesis of holey graphene‐supported single‐atom catalysts by laser irradiation,^[^ ^66^ ^]^ it is still hugely challenging to prevent particle aggregation and particle enlargement during the synthesis of carbon‐supported single‐atom catalysts (SACs),^[^ ^67^ ^]^ also indicated by the RuCu states of LUCA‐synthesized RuCu/rGO composites (Figure S12a,b, Supporting Information).

The HER performance of RuCu/CNTs composites changed with the loading of RuCu NPs with the Ru_95_Cu_5_/CNTs catalyst being the best, outperforming the Ru_97_Cu_3_/CNTs, Ru_89_Cu_11_/CNTs, Ru_20_Cu_80_/CNTs, and Ru@Cu/CNTs catalysts obtained using the conventional heating method (Figure S13, Supporting Information, Figure 5g). The LUCA‐synthesized Ru_89_Pd_11_ and Ru_86_Rh_14_ alloys (Figure S13, Supporting Information, and Figure 5h) also exhibited excellent HER performance with overpotentials of 21 and 22 mV, respectively, at a current density of 10 mA cm^−2^. Despite being inferior to Ru_95_Cu_5_/CNTs, they are still superior to commercial Pt/C. We also tested the influence of the irradiation period on the morphologies of the LUCA‐synthesized CNTs‐supported Ru‐based alloy catalysts to identify the general trend in their HER performance (Figure S14, Supporting Information). The HER performance strongly depended on the irradiation period, following the general trend of 30 min > 100 min > 5 min. After a short period of irradiation, the loading density of the Ru‐M alloy NPs was highly limited (Figure S15a,b, Supporting Information), so the HER performance was poor, whereas prolonging the irradiation period to 100 min caused the aggregation and enlargement of alloy particles (Figure S15c,d, Supporting Information), distinctively compromising the HER performance and highlighting the importance of the Ru‐M (M = Cu, Rh, and Pd) alloy size for high‐performance HER catalyst development. The smaller the alloy particle size, the more active sites are exposed to reactions at a specific shape‐defined mass of the catalyst, which can effectively reduce the cost of practical catalytic applications.

Ru_95_Cu_5_/CNTs also have robust long‐term HER stability. The LSV curve of the Ru_95_Cu_5_/CNTs catalyst shifted only slightly after 5000 CV cycles (Figure 5j), and its current density at a constant overpotential of 10 mA cm^−2^ remained unchanged for 40 h (Figure 5i). By comparison, the commercial 20% Pt/C catalyst exhibited ≈50% performance degradation after 5 h of testing (Figure S16, Supporting Information). TEM characterization showed that the Ru_95_Cu_5_ alloy NPs were still homogeneously distributed on the CNTs without distinct agglomeration and maintained their size at ≈3 nm, while severe aggregation occurred in the commercial Pt/C catalyst (Figure S17, Supporting Information), demonstrating the higher stability of the Ru_95_Cu_5_/CNTs catalyst in harsh environments where the CNTs play a crucial role in preventing NPs from aggregation.

The XPS spectra show that the Ru peak of the Ru_95_Cu_5_/CNTs shifts to a lower binding energy than that of the Ru/CNTs (Figure S18a, Supporting Information), whereas the Cu 2p spectrum shifts to a higher binding energy (Figure S18b, Supporting Information), indicating that Ru gained electrons from Cu.^[^ ^69^ ^]^ Changes in the electronic structures of Ru and Cu lead to changes in their d‐band filling, similar to the RuAu catalysts.^[^ ^59^ ^]^ For Ru, the d‐band filling increased as a result of electron gain, whereas for Cu atoms, the d‐band filling decreased after electron loss. According to the d‐band center theory, both changes affect H absorption.^[^ ^70^ ^]^ In addition to the alteration of the electronic structures induced by the Ru and Cu interaction, the surface charge distribution may also change, leading to the generation of more Cu^0^/^1+^ states, which may optimize the adsorption bonding of the active sites to the reaction species, improving HER performance.^[^ ^71^ ^]^ Our results are in accordance with previous studies that reported a volcano‐like relationship between electrocatalysts with varying Ru/Cu atomic ratios toward HER performance,^[^ ^72^ ^]^ which is attributed to the Sabatier principle with a proper surface electron structure state and H adsorption energy. A volcano HER performance trend was also observed for three‐dimensional nanoporous Cu‐Ru (np‐CuRu) alloys, where np‐Cu_53_Ru_47_ is superior to np‐Cu_88_Ru_12_ and np‐Cu_35_Ru_65_,^[^ ^11^ ^]^ indicating different case‐by‐case optimal Ru/Cu ratios.

The C 1s spectra (Figure S18c–e, Supporting Information) show that C─O groups exist on all catalysts, in accordance with the conditions of the Ru_13_─O_3_─C_60_ catalyst.^[^ ^73^ ^]^ The high‐resolution Ru 3p and Cu 2p XPS spectra (Figure S18a,b, Supporting Information) and XPS survey spectra (Figure S18f, Supporting Information) also indicate that Ru, RuCu, and Cu particles on the CNTs are all partially oxidized. Hence, strong interfacial Ru─O─C bonds should form in the LUCA‐synthesized Ru_95_Cu_5_/CNTs catalysts, rendering them highly stable against long‐term catalytic applications. The abundant Ru^0^ in the RuCu alloy particles is the main factor promoting the HER activity.^[^ ^74^ ^]^ The reason for the Ru─O─C bond could be that: laser irradiation of CNTs in ethanol damages the CNTs, decomposes ethanol molecules, and yields free oxygen species, which may oxidize the CNTs surfaces to form C─OH, allowing in situ bonding of Ru atoms for particle nucleation and RuCu alloying.

**Figure 6:**
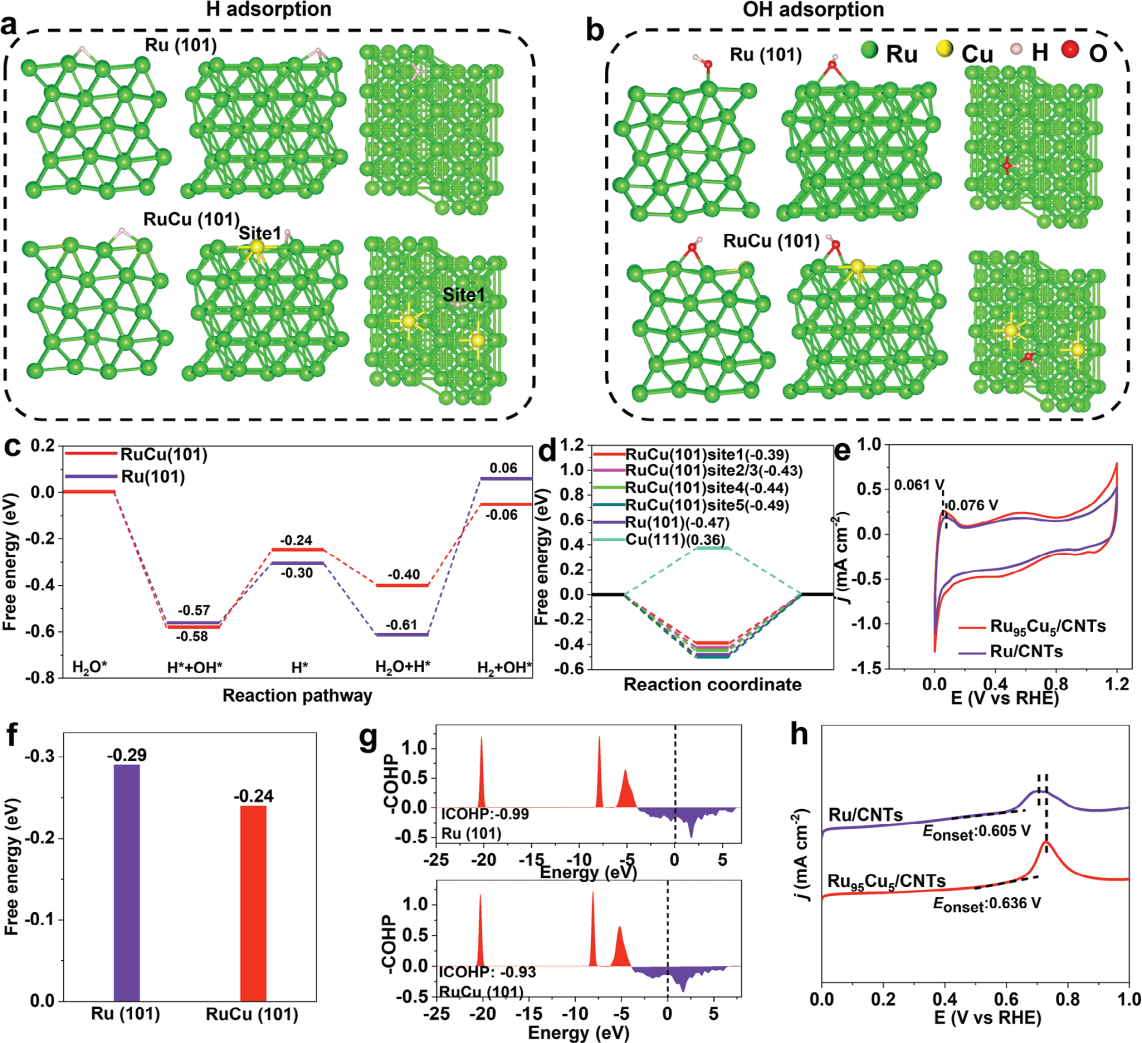
HER mechanism analysis. a,b) Theoretical models of H adsorption and OH adsorption on Ru (101) and Site‐1 of RuCu (101), respectively. Theoretical models without H adsorption, H adsorption on sites 2–5 of RuCu (101), and H adsorption on Cu (111) are shown in Figures S19–S21, Supporting Information, respectively. c) Free energy diagrams of the elementary steps in alkaline HER for Ru (101) and RuCu (101). (d) Adsorption free energies of H for 5‐site of RuCu (101), Ru (101), and Cu (111). e) CV curves of Ru_95_Cu_5_/CNTs, and Ru/CNTs. f) Adsorption free energy of OH for RuCu (101) and Ru (101). g) Projected crystal orbital Hamilton populations (COHP) between chemisorbed *O and active metal centers on Ru (101) and RuCu (101). h) CO stripping analysis of Ru_95_Cu_5_/CNTs and Ru/CNTs.

DFT modeling/calculations of the H/OH adsorption free energy and experimental measurements of the hydrogen binding energy (HBE) in the range of 0–1.0 V versus RHE in Ar‐saturated 1.0 m KOH were performed to obtain insights into the mechanism of the excellent alkaline HER performance of Ru_95_Cu_5_/CNTs catalysts. A model of Cu‐atom doped Ru NP was constructed with the relevant structures, as shown in **Figure** **6**a,b and Figures S19–S21 (Supporting Information). The selection of the (101) plane of Ru and RuCu for modeling/calculations was according to the HRTEM characterizations (Figure 4a; Figure S4a, Supporting Information), also referred from the RuCo alloys.^[^ ^75^ ^]^ It is calculated that the rapid dissociation of H_2_O into adsorbed OH and H can provide protons quickly for subsequent reactions. As shown in Figure 6c, the reaction free energy of the first H_2_O dissociation (H_2_O‐H+OH) are 0.57 and 0.58 eV on Ru and RuCu, respectively, while the reaction free energy of the second H_2_O dissociation (H_2_O+H‐H_2_+OH) to generate H_2_ are 0.67 and 0.34 eV on Ru and RuCu, respectively. This suggests that the introduction of Cu can accelerate the dissociation of water, providing more protons for the subsequent reaction. As an activity indicator for HER electrocatalysts, the H adsorption‐free energy largely influences the catalyst activity in alkaline solutions. RuCu(101) (−0.39 eV) on Site 1 exhibits a better H adsorption‐free energy than Ru(101) (−0.47 eV), Cu (111) (0.36), and RuCu(101) (−0.43–0.49 eV) on Sites 2–5, as shown in Figure 6d, indicating that Cu doping facilitates H adsorption/desorption, in accordance with the RuCo alloy alkaline HER catalysts.^[^ ^75^ ^]^ The under‐potential deposition of the hydrogen (*H*_upd_) desorption peak (*E*_peak_) is directly related to the HBE of active sites following the equation Δ*H* = −FE_peak_.^[^ ^76^ ^]^ As shown in Figure 6e, the *H*_upd_ peak of Ru_95_Cu_5_/CNTs (0.061 V) was lower than that of Ru/CNTs (0.076 V), indicating that the enhanced HER activity was related to weaker H chemisorption intensity, thus triggering accelerated H desorption behavior.

Strong OH adsorption has been reported to block the active sites of Ru, thus reducing the HER efficiency of Ru‐based catalysts, whereas weaker OH adsorption can alleviate the site‐blocking effect caused by OH adsorption, thus releasing more active sites for the HER.^[^ ^77^ ^]^ The adsorption‐free energies of OH on Ru (101) and RuCu (101) were calculated and are shown in Figure 6f. Compared with −0.29 eV for Ru (101), RuCu (101) exhibits a lower OH adsorption free energy of −0.24 eV, indicating that Ru_95_Cu_5_/CNTs can desorb OH much faster and accelerate the HER reaction more efficiently, which was also supported by crystal orbital Hamilton population (COHP) analysis of the interaction between the *O intermediate and the active metal centers, i.e., Ru on Ru (101) and Ru on RuCu (101), as shown in Figure 6g. The integrated COHP (ICOHP) which quantifies the bonding contribution, increases from −0.99 eV (Ru−*O bond in Ru (101)) to −0.93 eV (Ru−*O bond in RuCu (101)), revealing that the Ru−*O bond in Ru (101) is slightly stronger than that of Ru−*O in RuCu (101). This indicates that *OH desorption occurred much more readily on RuCu (101). Hence, the overpotential of LUCA‐Ru_95_Cu_5_/CNTs was reduced to 17 mV from 49 mV for Ru/CNTs. As interfacially adsorbed OH can promote the oxidation of adsorbed CO (CO_ad_) intermediates to CO_2_, CO stripping tests are often used to evaluate the OH adsorption capacity of electrocatalysts.^[^ ^78^ ^]^ As shown in Figure 6h, the CO oxidation initial onset and peak potentials of Ru_95_Cu_5_/CNTs positively shift compared with those of Ru/CNTs, indicating that the adsorption of Ru−OH is weaker for Ru_95_Cu_5_/CNTs than for Ru/CNTs, which is beneficial for the exposure of more active sites for the alkaline HER.

In light of the outstanding HER performance of Ru_95_Cu_5_/CNTs in 1.0 m KOH, a two‐electrode system was also built to evaluate the overall water‐splitting performance. Ru_95_Cu_5_/CNTs and RuO_2_ were employed as the cathode and anode, respectively. The polarization curves shown in Figure S22 (Supporting Information) indicate that the Ru_95_Cu_5_/CNTs||RuO_2_ catalysts are still better than 20%Pt/C||RuO_2_ for overall water splitting because they require a lower cell voltage of 1.77 V than 20%Pt/C||RuO_2_ (1.79 V) to deliver a current density of 10 mA·cm^−2^ in 1.0 m KOH, inferring their potential application for overall water splitting,^[^ ^79^ ^]^ which will be further studied in the future.

## 3 Conclusion

In summary, LUCA was demonstrated to be a facile platform for synthesizing robust CNTs‐supported sub‐5 nm dominant Ru‐M (M = Cu, Rh, and Pd) alloy NPs owing to its unique ultrafast heating/quenching processes, overcoming the limit/difficulty of thermodynamic immiscibility for alloying and spontaneous enlargement. The HER performances of the Ru_95_Cu_5_/CNTs composites are excellent, with an ultralow overpotential (17 mV) at 10 mA cm^−2^, much better than that of the 20% Pt/C catalyst (36 mV). In addition, the ultrahigh turnover frequency (42.2 H_2_ s^−1^) and mass activity (10.7 A mg^−1^) at −0.1 V (vs RHE) in 1.0 m KOH are 9.4‐ and 15.3‐fold higher than that of the 20% Pt/C catalyst, indicating their great potential as competitive high‐performance catalysts for sustainable applications. Both DFT analysis and experimental studies (CV curves, CO stripping) on the Ru_95_Cu_5_/CNTs and Ru/CNTs catalysts showed that laser ultrafast miscible alloying of Cu with Ru can optimize the Ru−H/OH binding energy to dramatically enhance the HER activity. This study provides a basis for the design of advanced small immiscible alloys and broadens their electrocatalytic applications.

## 4 Experimental Section

### Materials, Synthesis, Electrochemical measurements and simulation

RhCl_3_, RuCl_3_, Na_2_PdCl_4_, Cu(CH_3_COO)_2_, ethanol, and KOH were purchased from Sinopharm Chemical Reagent Co. Ltd.Nafion solution (5%) was purchased from Sigma‐Aldrich. The deionized water in the experiment was always ultrapure water (18.2 MΩ·cm). GO was purchased from Suzhou Hengqiu Technology Co. Ltd. High purity Cu target (99.99%) was acquired from Hefei Zhongke Napu New Material Co. Ltd. Nafion membrane (N115) adopted for overall water splitting was acquired from Dupont Co.Ltd.

### RuM (M = Cu, Pd, and Rh) composites synthesis by LUCA

5 mg of CNTs, 2 mg RuCl_3_, and x mg (where x = 0.5, 1, 3, and 30) Cu(CH_3_COO)_2_ were added to the bottle, and then 15 mL ethanol was put into the bottle. Then, the mixture was stirred for 10 h. Finally, the mixture was irradiated for 30 min by a fundamental Nd:YAG laser (355 nm) with a 20 Hz pulse repetition rate, 7 ns pulse duration, and 30 mJ per pulse laser energy. The obtained catalysts were denoted as Ru_97_Cu_3_/CNTs, Ru_95_Cu_5_/CNTs, Ru_89_Cu_11_/CNTs, and Ru_20_Cu_80_/CNTs. The synthesis of Ru_86_Rh_14_/CNTs and Ru_89_Pd_11_/CNTs was similar to that of Ru_95_Cu_5_/CNTs, except that the added Cu(CH_3_COO)_2_ was replaced by 0.25 mg RhCl_3_ or 0.3 mg Na_2_PdCl_4_. The synthesis of the Ru/CNTs catalyst was the same as the Ru_95_Cu_5_/CNTs, except that Cu(CH_3_COO)_2_ was not added. The Cu/CNTs catalyst was synthesized in a similar way to Ru_95_Cu_5_/CNTs, where only 30 mg of Cu(CH_3_COO)_2_ was added, but without any RuCl_3_. To show the influence of the irradiation period on the alloy states and HER performances, 5 min and 100 min LUCA were also explored.

### Synthesis of RuCu/rGO composites by LUCA

GO (5 mg), 2 mg RuCl_3_, and 1 mg Cu(CH_3_COO)_2_ were added to a bottle, and then 15 mL ethanol was added. The mixture was stirred for 10 h. Finally, the salt mixture was irradiated for 30 min by a Nd: YAG laser (355 nm) with a 20 Hz pulse repetition rate, 7 ns pulse duration, and 30 mJ per pulse laser energy.

### Synthesis of Ru@Cu/CNTs by conventional heating method

20 mg CNTs, 16 mg RuCl_3_, and 8 mg Cu(CH_3_COO)_2_ were added into a glass bottle with a volume of 30 mL, and then 15 mL of ethanol and 5 mL of deionized water were added into the bottle for ultrasonic dispersion, and after the end of the ultrasonication, it was stirred for 10 h. After the end of the stirring, it was freeze‐dried. After drying, the black powder was calcined in a tube furnace with 5% H_2_/Ar, and the temperature was increased to 600 °C for 4 h with a temperature elevation rate of 5 °C per min. After calcination, the samples obtained were termed as Ru@Cu/CNTs.

### Synthesis of Cu NPs and Cu/CNTs Composites By Femtosecond Laser Ablation

A Cu target was immobilized in a vessel containing 15 mL of ethanol. The liquid thickness between the sample surface to the air‐liquid interface was ≈2 cm. The Cu target was then ablated using a focused femtosecond (35 fs) laser (800 nm) for 15 min at a power of 400 mW and a pulse repetition rate of 1000 Hz. An area of 5mm*5 mm was scanned with a spot size of 55 µm. The scan speed was set at 3 mm s^−1^. Finally, the Cu nanoparticles were obtained. Cu/CNTs were synthesized in the same steps as Cu nanoparticles, except that 15 ml of ethanol was replaced with a mixture of 5 mg of CNTs and 15 mL of ethanol.

### Characterization

The morphologies and structures of samples were characterized by transmission electron microscopy (TEM) on a JEM‐ARM300F with a spherical aberration corrector. Powder X‐ray diffraction (XRD) spectra were recorded on a Rigaku X‐ray diffractometer with Cu Kα radiation (*λ =* 0.15419 nm). The composition in the catalyst suspension was estimated through inductively coupled plasma optical emission spectrometry (ICP‐OES). The surface chemical contents of the products were analyzed by X‐ray photoelectron spectroscopy (XPS, Thermo ESCACLB 250). The H_2_ was tested by using Gas chromatography (FULI INSTRUMENTS: GC9720Plus). For quantification of the noble metal loading, the catalyst was first weighed, then the weighed catalyst was added into aqua regia and was dissolved with a microwave dissolver, after dissolution, the dissolved solution was filtered and it was diluted to a reasonable concentration, and finally tested the content of each element in the diluted solution with ICP to finally arrive at the noble metal loading.

### Electrochemical HER Measurements

Electrochemical tests were performed on the CHI 660E electrochemical workstation in a conventional three‐electrode system, with a Hg/HgO electrode as the reference electrode and a carbon rod as the counter electrode. All potentials mentioned below were based on reversible hydrogen electrodes (RHE). The formula for potential conversion was E(V vs RHE) = E (V vs Hg/HgO) + 0.098 + 0.059 × pH. The working electrodes for HER were made by applying catalyst ink onto the glassy carbon electrode (GCE) with an area of 0.07065 cm^−2^. The catalyst inks were prepared by dispersing 2 mg powder in a solution containing 1 mL of isopropanol and 10 µL of 5 wt.% Nafion solution by sonication for 0.5 h. Then 10 µL ink was dropped on the GCE for drying in the air, and the loading mass of the catalyst was 0.2802 mg cm^−2^. The HER tests were carried out in an Ar‐saturated 1.0 m KOH solution. Linear sweep voltammetry (LSV) was used to test the HER activity of the catalyst with a scan rate of 5 mV s^−1^. CV measurements were performed for over 100 cycles (scan rate: 20 mV s^−1^) to reach a stable state. Then, CV curves were recorded with a scan rate of 5 mV s^−1^. Electrochemical impedance spectroscopy (EIS) was carried out at the open circuit with a frequency from 0.01–100 KHz. All LSV curves were corrected with iR‐compensation, and the compensation level was 90%. The ECSA was evaluated by underpotential deposition of Cu (Cu‐UPD, 420 µC cm^−2^). Then the intrinsic activity was calculated by ECSA. The mass activity was calculated based on the loading of noble metals. TOF values were calculated by the following equation: TOF = I/2Fn, where I was the current (A), F was the Faraday constant (96485.3 C mol^−1^), and n was the number of active sites (mol). The factor 1/2 represents the transfer of two electrons for one hydrogen molecule generation. The n can be calculated from the charge accumulation (Q) of Cu‐UPD following the equation: *n* = Q_Cu_/2F. CO stripping experiments with a scan rate of 20 mV s^−1^ were performed to reflect the OH binding ability of samples.

### Electrochemical Overall Water Splitting Measurements

The overall water splitting tests were conducted in a two‐electrode system (Figure S) which was assembled by the as‐synthesized materials both as cathode and anode. As for the Ru_95_Cu_5_/CNTs||RuO_2_ and Pt/C||RuO_2_, 3 mg corresponding catalysts were dissolved in the mixture of 20 µL 5 wt.% Nafion solution and 1 mL isopropanol, after a sonication treatment for 30 min, a homogeneous catalyst ink was formed. Then 200 µL of catalyst ink was dropped onto 1 cm ×1 cm carbon paper and dried in air.

### Computation

The spin‐polarized density functional theory (DFT) calculations were performed based on the generalized gradient approximation (GGA) implemented in the VASP 5.4.4 package.^[^ ^80^ ^]^ Electronic exchange and correlation were described by Perdew−Burke−Ernzerhof (PBE) functional.^[^ ^81^ ^]^ All‐electron plane‐wave basis sets with the projector augmented wave (PAW) potentials were adopted and the cutoff energy was set to be 450 eV. A dense enough k‐point sampling was checked with energy tolerance in 1 meV per atom. The surfaces were represented by periodic slab models. A vacuum >12 Å thick was inserted in each model to avoid interaction with imaging‐free surfaces. Similarly, the lattice parameters of each slab supercell were also >10 Å to avoid interaction between the adsorbed molecular and its image. The optB88‐vdW functional was used to calculate the adsorption energy, which was an efficient method to approximately account for the long‐range vdW interaction.^[^ ^82^ ^]^ The hydrogen adsorption free energy Δ*G*_H*_ = *E*_(surf+H)_ − *E*_(surf)_ − 1/2*E*_(H2)_+ Δ*E*_ZPE_ − *T*ΔS, where Δ*E*_ZPE_ and Δ*S* were the difference in the zero‐point energy and entropy between the adsorbed H atom and the gaseous phase H_2_.

## Conflict of Interest

The authors declare no conflict of interest.

## Supporting information



Supporting Information

## Data Availability

The data that support the findings of this study are available from the corresponding author upon reasonable request.
