# Supplementary material for: Laser Ultrafast Confined Alloying of Sub‐5 nm RuM (M = Cu, Rh, and Pd) Particles on Carbon Nanotubes for Hydrogen Evolution Reaction
Source: Adv Sci (Weinh). 2025 Feb 21;12(19):2415065. doi: 10.1002/advs.202415065 (PMC12097106; doi:10.1002/advs.202415065)
Supplement: Supplementary file 1 — Supporting Information [file ADVS-12-2415065-s001.docx]

Supporting Information

Laser Ultrafast Confined Alloying of Sub-5 nm RuM (M = Cu, Rh, and Pd) Particles on Carbon Nanotubes for Hydrogen Evolution Reaction

Taiping Hu^1,2^, Dongshi Zhang ^3,*^, Ningning He^1,2^, Shuxian Wei^1,2^, Xingyu Kang^1,2^, Wei Zhang^4^, Yunyu Cai^1^, Yixing Ye^1^, Pengfei Li^1*^, Changhao Liang^1,2,5*^

^1^Key Laboratory of Materials Physics and Anhui Key Laboratory of Nanomaterials and Nanotechnology, Institute of Solid State Physics, Chinese Academy of Sciences, Hefei 230031, P. R. China

^2^Department of Materials Science and Engineering, University of Science and Technology of China, Hefei 230026, P. R. China

^3^Shanghai Key Laboratory of Materials Laser Processing and Modification, School of Materials Science and Engineering, Shanghai Jiao Tong University, Shanghai 200240, China

^4^Institute for Energy Research, Jiangsu University, Zhenjiang 212013, PR China

^5^Lu’an Branch, Anhui Institute of Innovation for Industrial Technology, Lu’an 237100, China

Emails: [zhangdongshi@sjtu.edu.cn](mailto:zhangdongshi@sjtu.edu.cn), [pfli@issp.ac.cn](mailto:pfli@issp.ac.cn), chliang@ issp.ac.cn


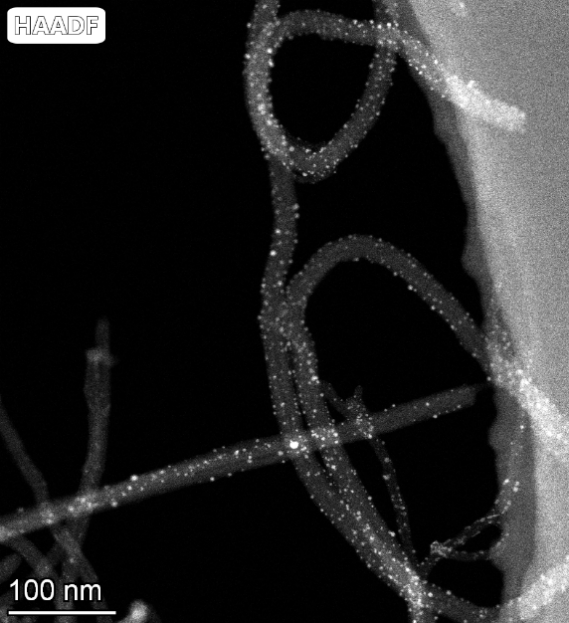

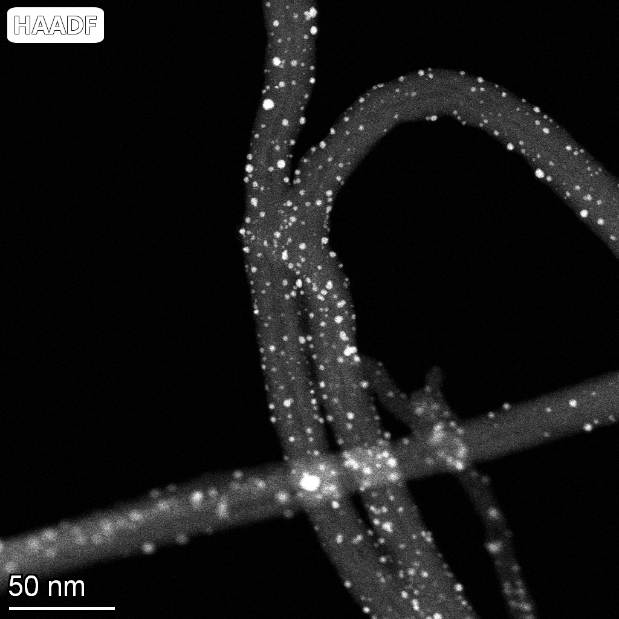


**Figure S1.** TEM images of Ru_95_Cu_5_/CNTs composites synthesized by LUCA.


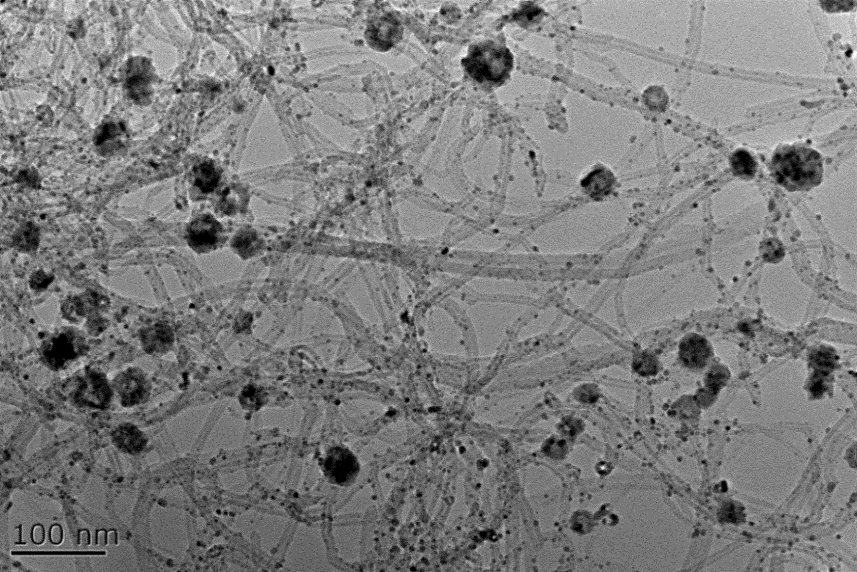


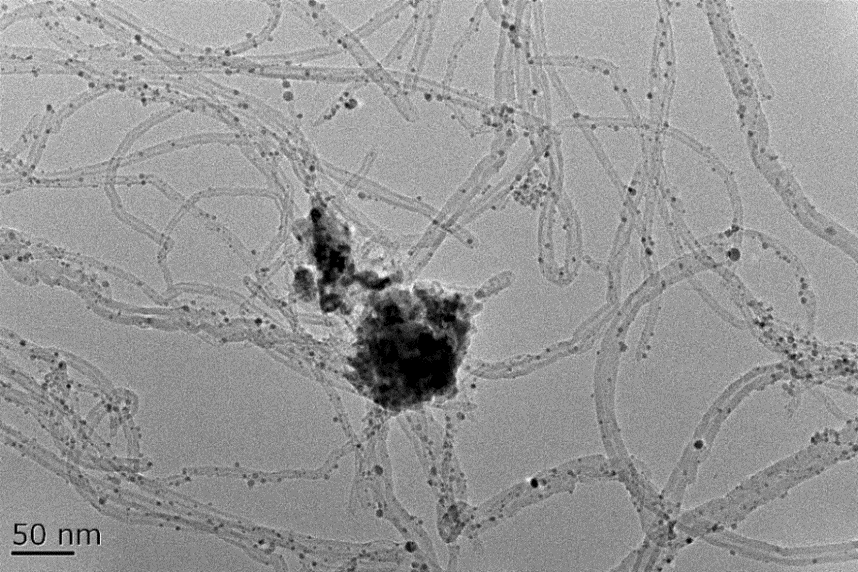


**Figure S2.** TEM images of Ru@Cu/CNTs composites synthesized by conventional heating method, showing severe NPs aggregation and CNTs linking.

**Figure S3.** XRD spectrum of Ru@Cu/CNTs composites synthesized by conventional heating method.


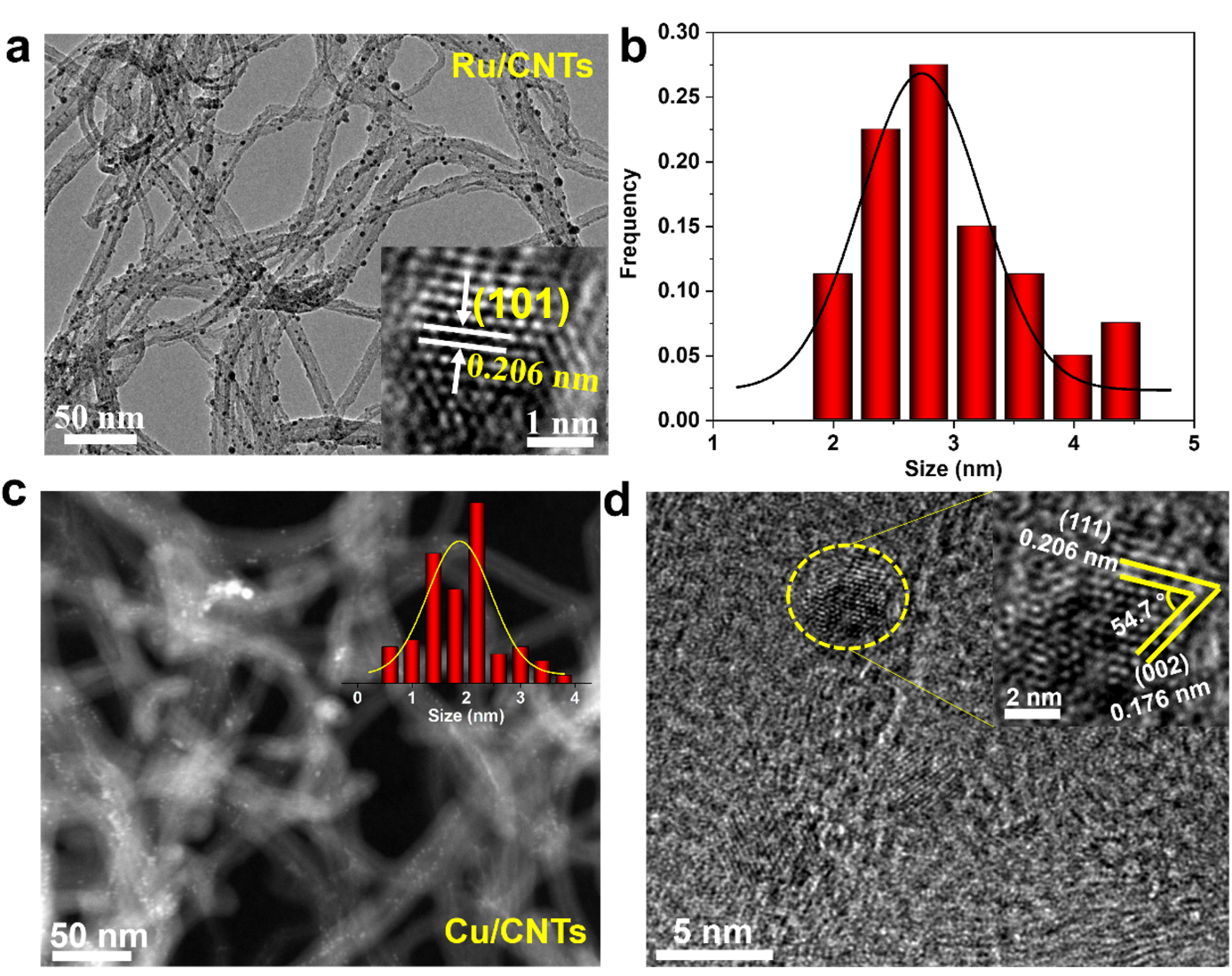


**Figure S4.** (a) TEM image of Ru/CNTs catalyst (inset: HRTEM image of a Ru NP). (b) Size distribution of Ru NPs. (c, d) TEM and HRTEM images of Cu/CNTs catalyst.

**Figure S5.** XRD spectra of CNTs and LUCA-synthesized CNTs-supported Ru NPs and Ru-based alloys obtained by LUCA.


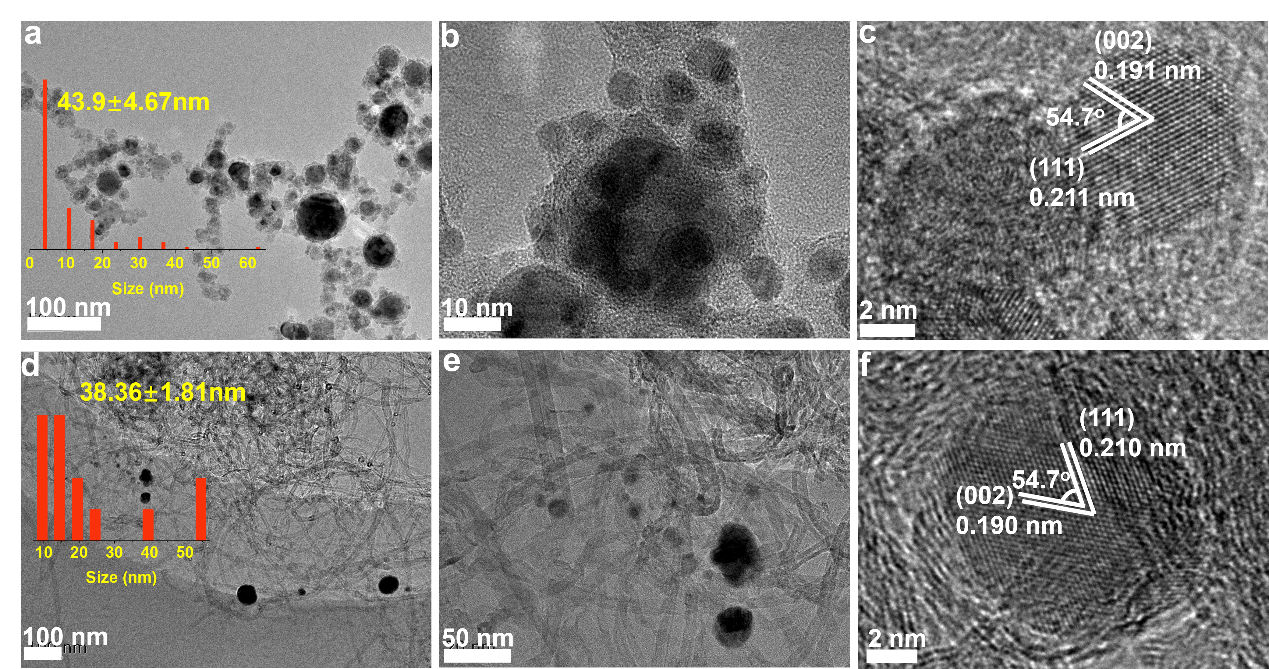


**Figure S6.** Fs laser ablation of Cu in ethanol and ethanol-CNTs solutions. (a-c) TEM images of Cu particles (inset: size distribution of Cu NPs), (c) HRTEM image of Cu. (d-e) TEM images of Cu/CNTs, (f) HRTEM image of Cu. The measured fringe spacing values are 0.211/0.210 and 0.191/0.190 nm, which correspond to the (1 1 1) and (0 0 2) crystalline planes of face-centered cubic Cu, respectively. The measured angle (54.7°) between this two crystalline planes matches well with the theoretical value.


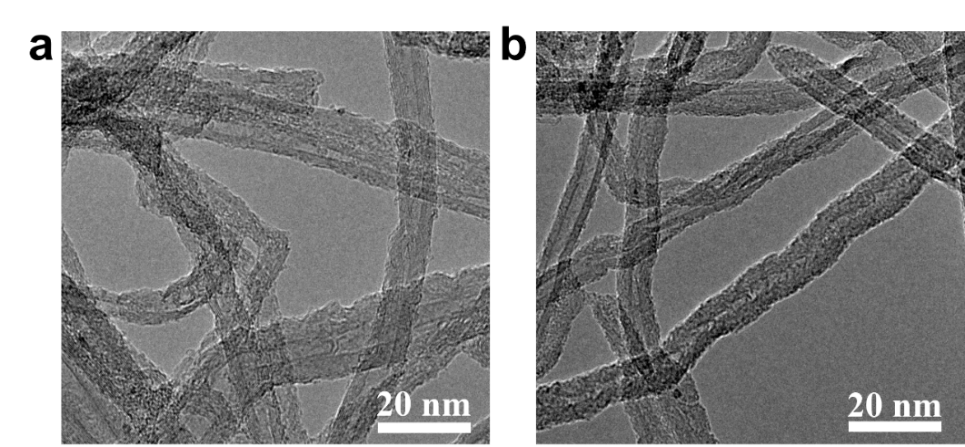


**Figure S7.** Complete dissolution (mere appearance of CNTs) of metals in aqua regia **(**a-b) TEM images of Ru_95_Cu_5_/CNTs and Ru/CNTs, respectively.

**Table S1. The overpotentials of Ru_95_Cu_5_/CNTs and other references.**

| **Catalyst** | **Electrolyte** | **(*η*_10_) Overpotential (mV)** | **Tafel Slope**  **(mV dec^-1^)** | **Morphology (size)** | **Ref.** |
| --- | --- | --- | --- | --- | --- |
| **Ru_95_Cu_5_/CNT** | **1.0 KOH** | **17** | **28.4** | **Nanoparticles (3 nm)** | **This work** |
| **RuAu SAAs** | **1.0 KOH** | **24** | **37** | **Nanoparticles (8 nm)** | **Adv. Energy Mater. 2019, 9, 1803913.** |
| **P-Ru/C** | **1.0 KOH** | **31** | **105** | **Nanoparticles (3 nm)** | **ACS Catal. 2020, 10, 11751−11757.** |
| **RuAu/RGO** | **1.0 KOH** | **56** | **113** | **Nanoparticles (2 nm)** | **Chem. Eng. J. 2021, 421, 129987.** |
| **Sr_2_RuO_4_** | **1.0 KOH** | **61** | **51** | **Bulk ( / )** | **Nat. Commun. 2019, 10, 149.** |
| **RuBe** | **1.0 KOH** | **34.8** | **28.9** | **Nanosheet (few micrometers )** | **Chem. Eng. J. 2021, 421, 129741.** |
| **Au-Ru-2 NWs** | **1.0 KOH** | **50** | **30.8** | **Nanowire@nanorod ( / @3.7±0.8 nm)** | **Nat. Chem. 2018, 10, 456-461.** |
| **RP-CPM** | **1.0 KOH** | **24** | **47.3** | **Nanoparticles (12 nm)** | **Sci. Adv. 2020, 6, eabb4197.** |
| **V-SRCO** | **1.0 KOH** | **57.8** | **35** | **Nanoparticles (few nm)** | **Adv. Energy Mater. 2023, 13, 2301779.** |
| **Ru-Mo_2_C/CN** | **1.0 KOH** | **34** | **80** | **Nanoparticles (1.8 nm)** | **J. Catal. 2020, 392, 313–321.** |
| **Ni@Ni_2_P-Ru** | **1.0 KOH** | **31** | **41** | **Nanoparticle@nanorod (4 nm@18-400nm)** | **J. Am. Chem. Soc. 2018, 140, 2731−2734.** |
| **UP-RuNi_SAs_/C** | **1.0 KOH** | **9** | **37.6** | **Agglomerate-like particles@nanoparticle (2μm@2nm)** | **Nat. Commun. 2024, 15, 2218.** |
| **np/Pt_1_Ru_1_-Ni_0.85_Se** | **1.0 KOH** | **46** | **32.4** | **Three-dimensional bicontinuous nanoporous structure ( / )** | **Small 2024, 20, 2311178.** |
| **Ordered Ru-Ni** | **1.0 KOH** | **23** | **25.9** | **Nanoparticles (3.3 nm)** | **Chem. Eng. J. 2024, 487, 150457.** |
| **Ni_91_Ru_9_@NC-600(mix)** | **1.0 KOH** | **27** | **42** | **Nanoparticles (6-10 nm)** | **Chem. Eur. J. 2023, 29, e202300062.** |
| **RuCo alloy** | **1.0 KOH** | **17** | **42** | **Spherical particles ( / )** | **ACS Appl. Energy Mater. 2024, 7, 4030−4039.** |
| **Ru_90_Ni_10_/rGOP** | **1.0 KOH** | **6** | **26** | **Nanoparticles (3 nm)** | **Small 2024, 2311509.** |
| **PtRu/mCNTs** | **1.0 KOH** | **15** | **33.5** | **Nanoparticles (2.09 nm)** | **Energy Environ. Sci.,**  **2022, 15, 102.** |
| **RuCuO_x_/NC** | **1.0 KOH** | **29** | **57.7** | **Polygonal nanoparticle @nanoparticle composite ( / )** | **Appl. Catal. B: Environ. 2023, 324, 122169.** |
| **CuRu-CNTs** | **1.0 KOH** | **39** | **89** | **Nanoparticles (4.3 nm)** | **J. Alloy. Compd. 2023, 936, 168349.** |
| **RhRu-MPSs** | **1.0 KOH** | **25** | **47.5** | **Spherical particles (56 nm)** | **ACS Appl. Mater. Interfaces 2021, 13, 5052−5060.** |

**Figure S8.** EIS plots of Ru_95_Cu_5_/CNTs and Ru/CNTs.

**
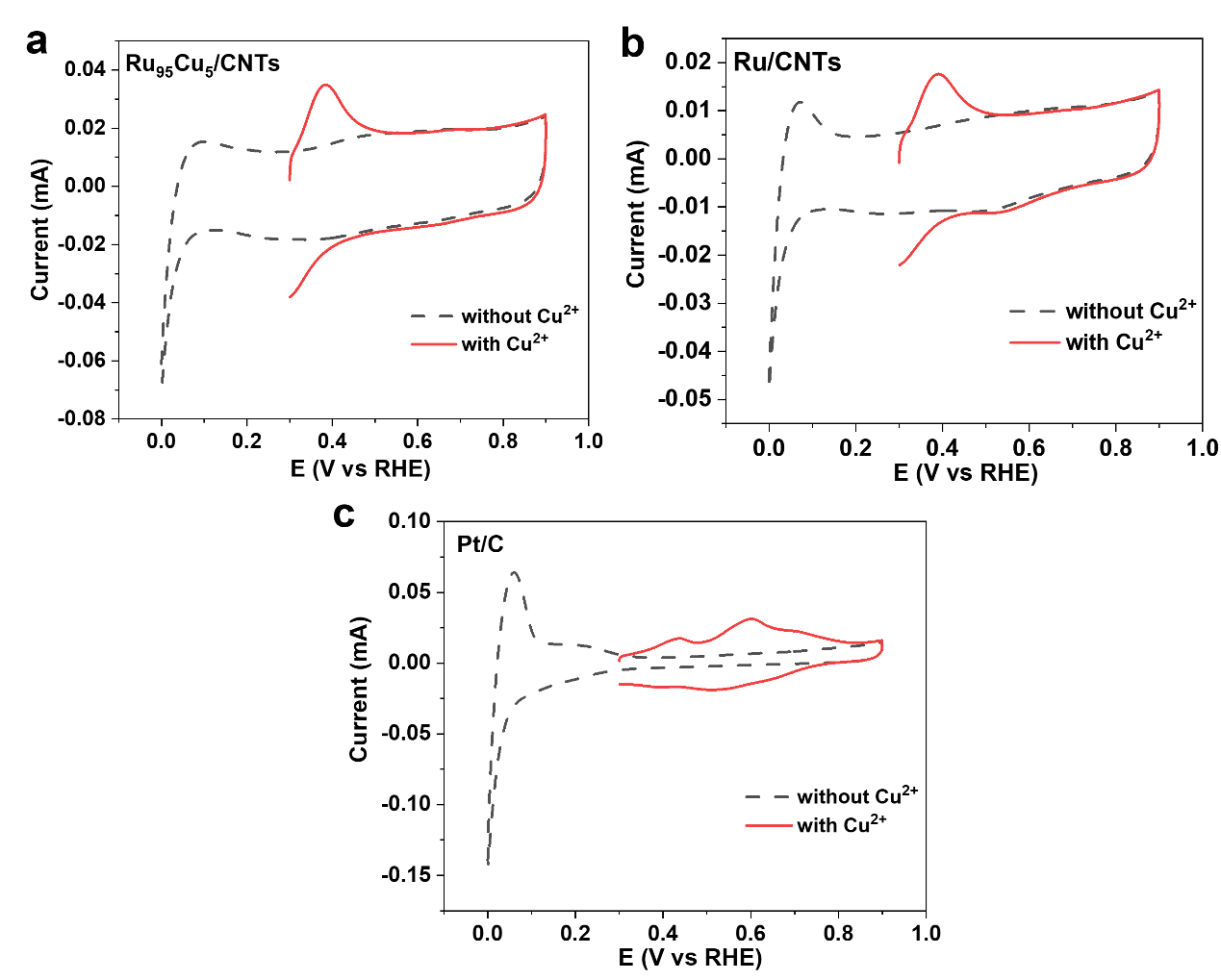
**

**Figure S9.** Cu-UPD curves of (a) Ru_95_Cu_5_/CNTs, (b) Ru/CNTs, and (c) Pt/C measured in 0.1 M H_2_SO_4_ + 2 mM CuSO_4_ with the scan rate of 10 mV s^-1^.


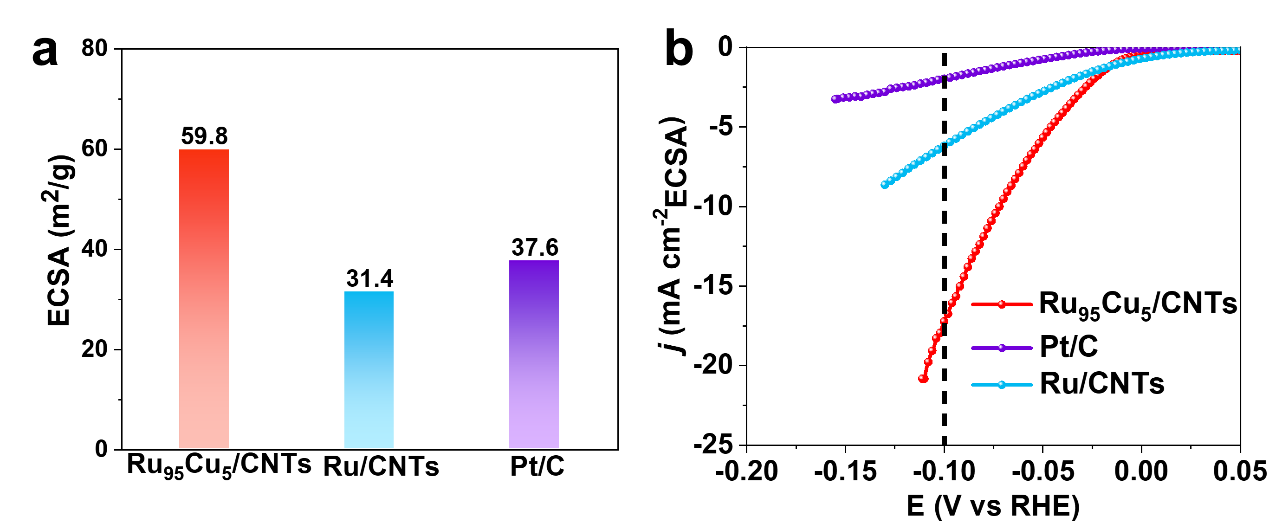


**Figure S10.** (a) ECSA values of Ru_95_Cu_5_/CNTs and control catalysts of Ru/CNTs and Pt/C. (b) ECSA-normalized LSV curves of Ru_95_Cu_5_/CNTs and control catalysts.


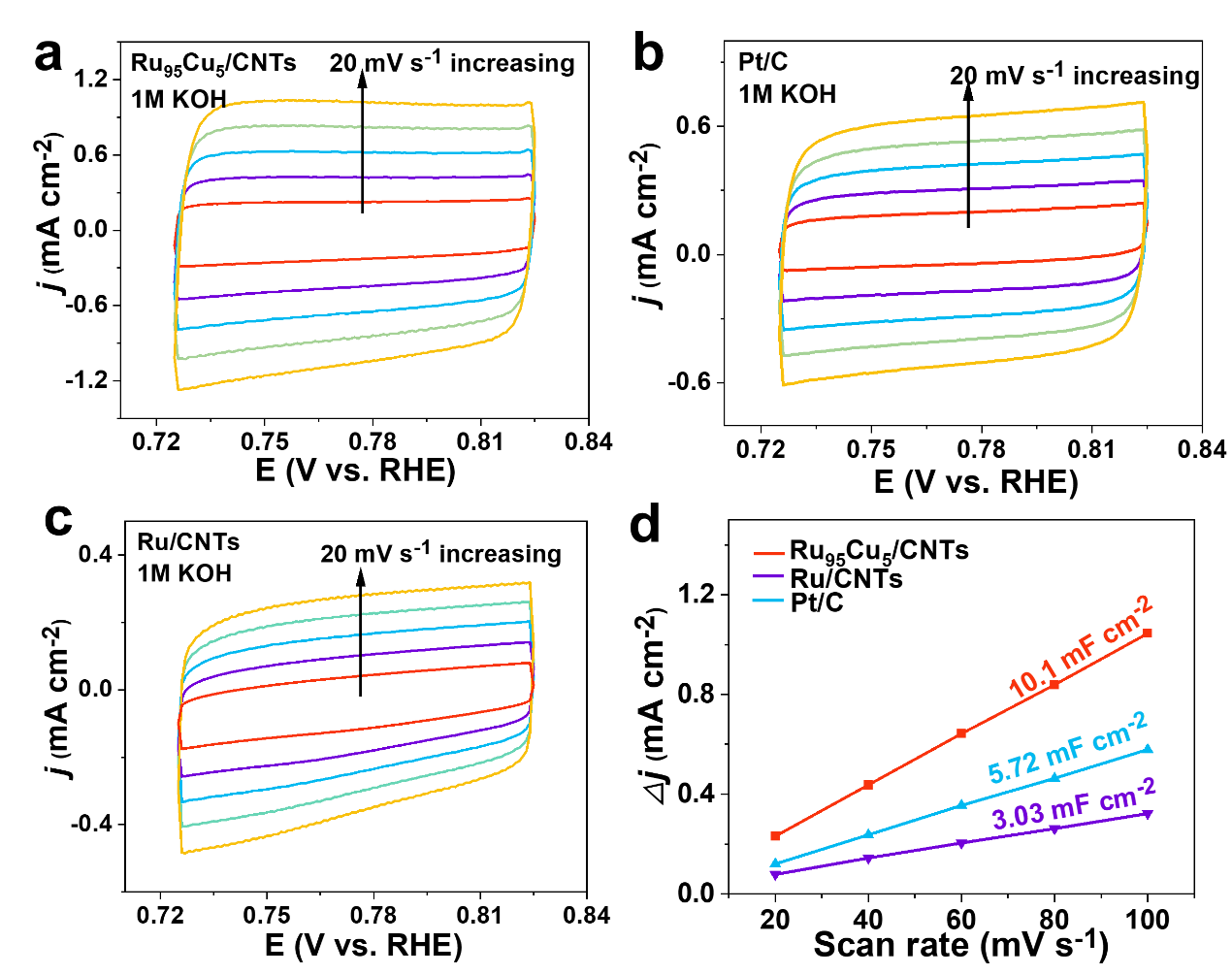


**Figure S11.** Cyclic voltammetry curves at scan rates ranging from 20 to 100 mV s^−1^ for (a) Ru_95_Cu_5_/CNTs, (b) 20% Pt/C, and (c) Ru/CNTs in 1.0 M KOH. (d) The capacitive current density versus scan rate for Ru_95_Cu_5_/CNTs, 20% Pt/C, and Ru/CNTs.


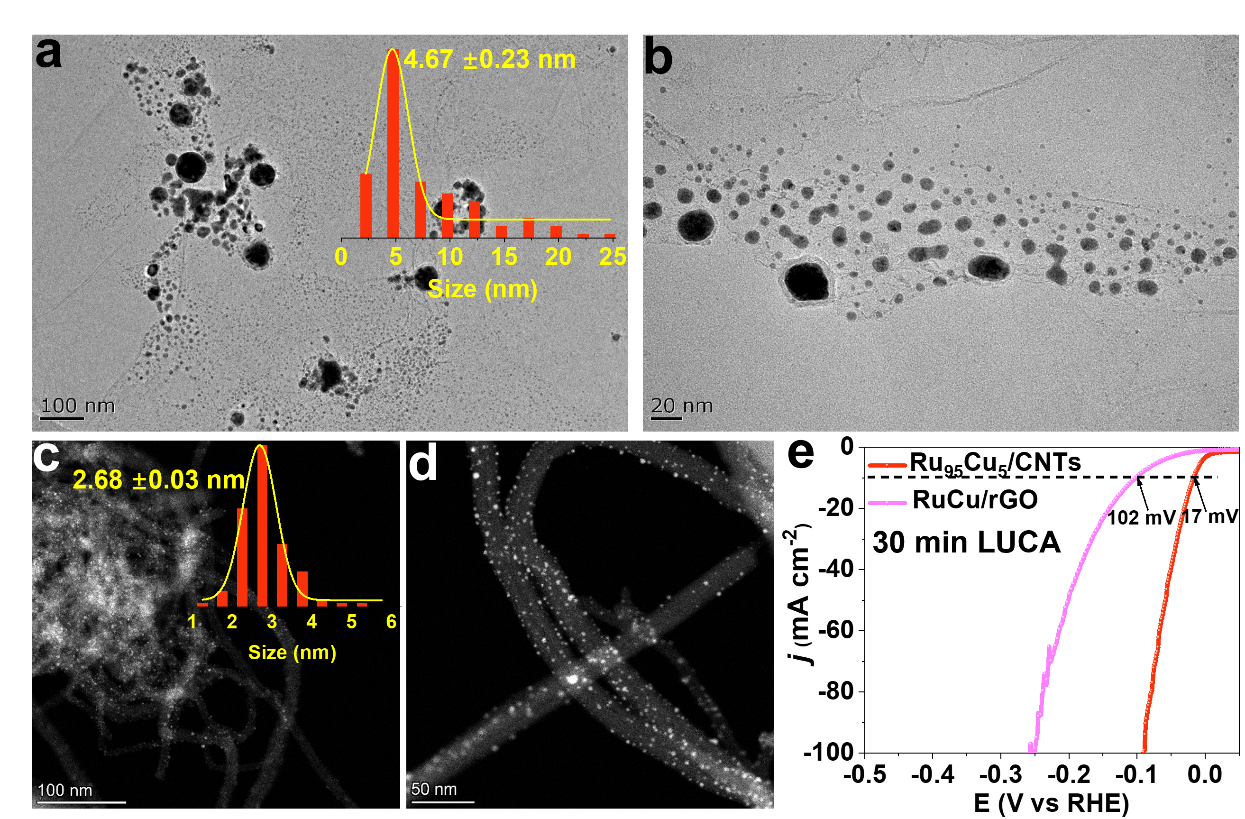


**Figure S12.** (a-b) TEM images of RuCu/rGO composites (inset: size distribution of RuCu NPs) synthesized by LUCA under the same condition with that of Ru_95_Cu_5_/CNTs. (c-d) TEM images of Ru_95_Cu_5_/CNTs (inset: size distribution of Ru_95_Cu_5_ NPs). (e) Alkaline HER performance of Ru_95_Cu_5_/CNTs and RuCu/rGO and the overpotential values at 10 mA cm^-2^ in Ar-saturated 1.0 M KOH condition.


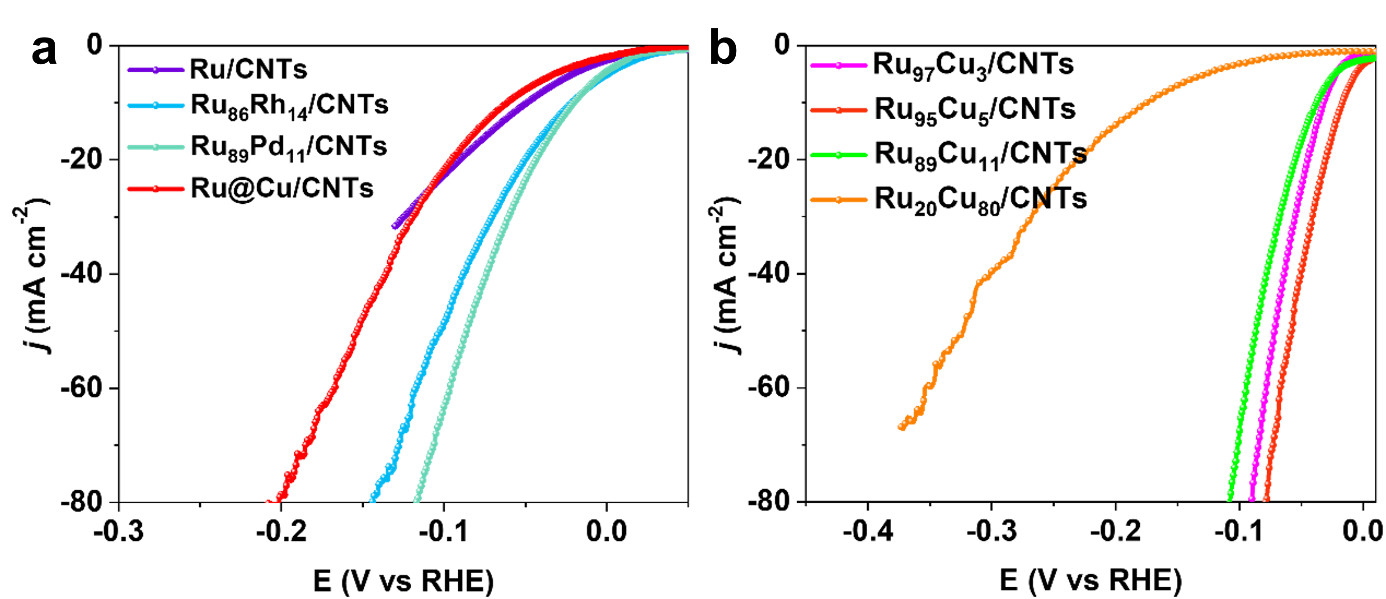


**Figure S13.** (a) HER polarization curves of different as-prepared Ru-based catalysts. (b) HER polarization curves of RuCu/CNTs with different amounts of Cu.


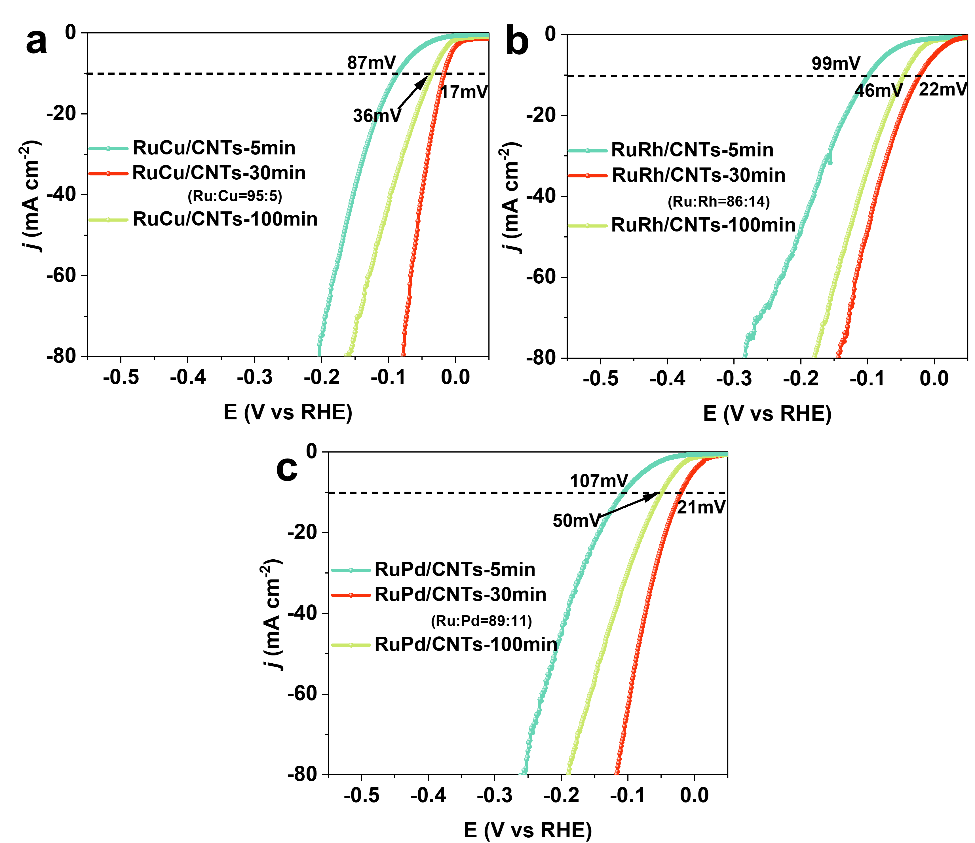


**Figure 14.** LSV curves of Ru_95_Cu_5_/CNTs, Ru_86_Rh_14_/CNTs, and Ru_89_Pd_11_/CNTs catalysts prepared by 5, 30 and 100 min. Corresponding overpotentials at a current density of 10 mA cm^-2^ are marked near the curves.


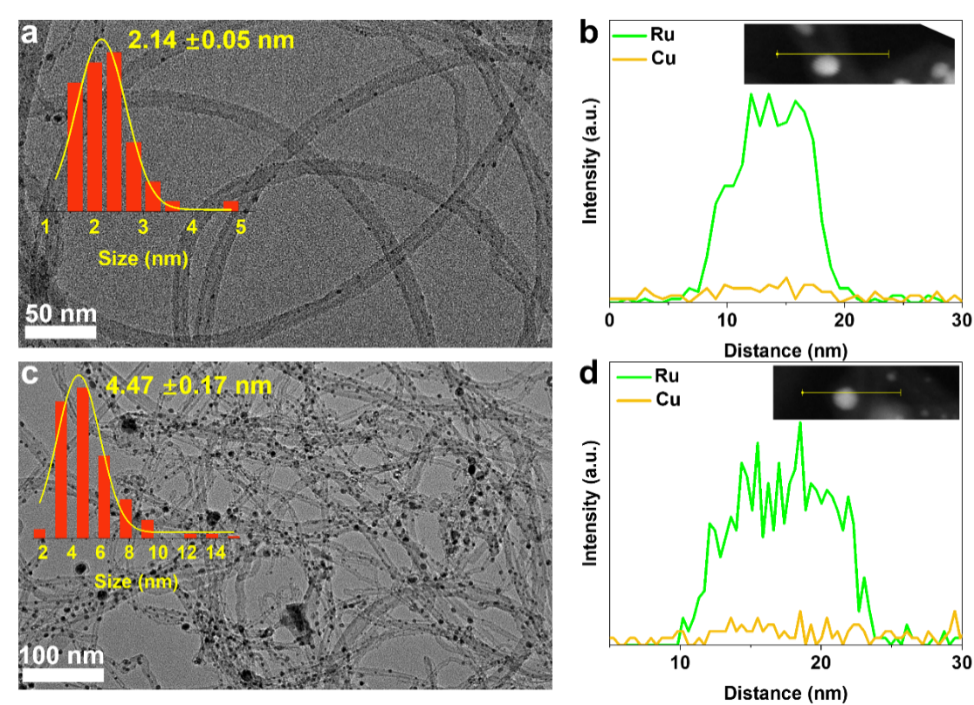


**Figure S15.** (a-b, c-d) TEM images and EDS line scanning profile of Ru_95_Cu_5_/CNTs-5min (inset: size distribution of RuCu NP) and Ru_95_Cu_5_/CNTs-100 min, (inset: HAADF-STEM image showing the line scanned).

**Figure S16.** Long-term *i-t* test of Pt/C catalyst for stability evaluation.

**
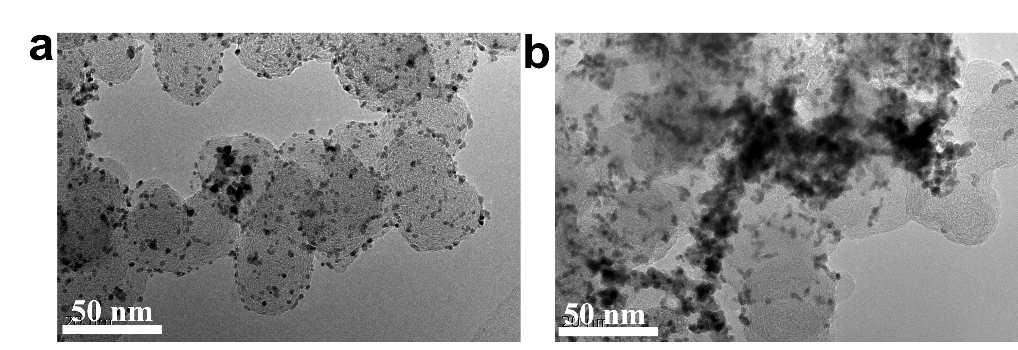
**

**Figure S17.** (a) TEM image of Pt/C catalyst before stability evaluation. (b) TEM images of Pt/C catalyst after stability evaluation.


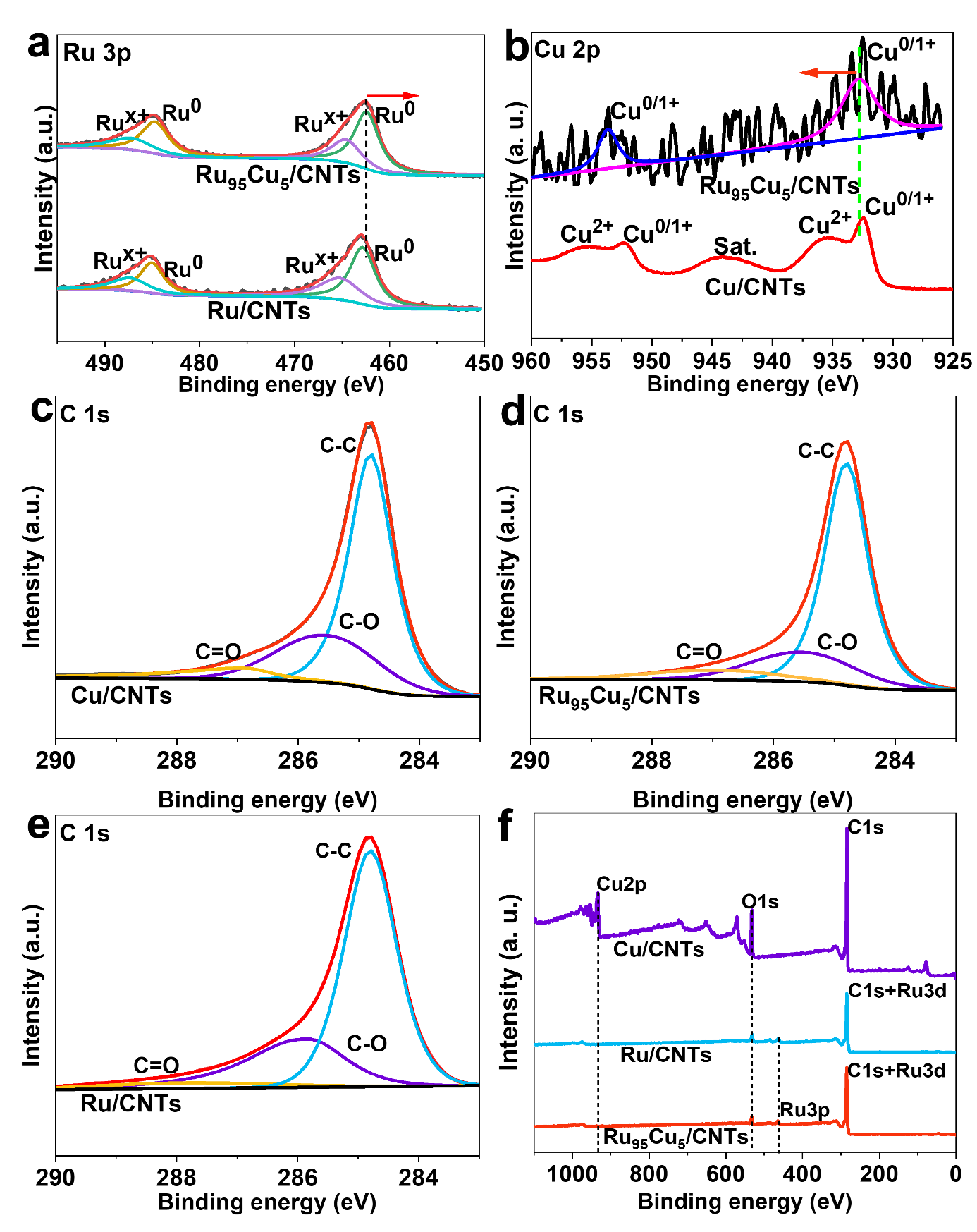


**Figure S18.** (a) High-resolution Ru 3p XPS spectra of Ru_95_Cu_5_/CNTs and Ru/CNTs. (b) High-resolution Cu 2p XPS spectra of Ru_95_Cu_5_/CNTs and Cu/CNTs. (c-e) High-resolution C 1s XPS spectra of Cu/CNTs, Ru_95_Cu_5_/CNTs and Ru/CNTs, respectively. (f) XPS survey specta of Ru_95_Cu_5_/CNTs, Ru/CNTs, and Cu/CNTs.


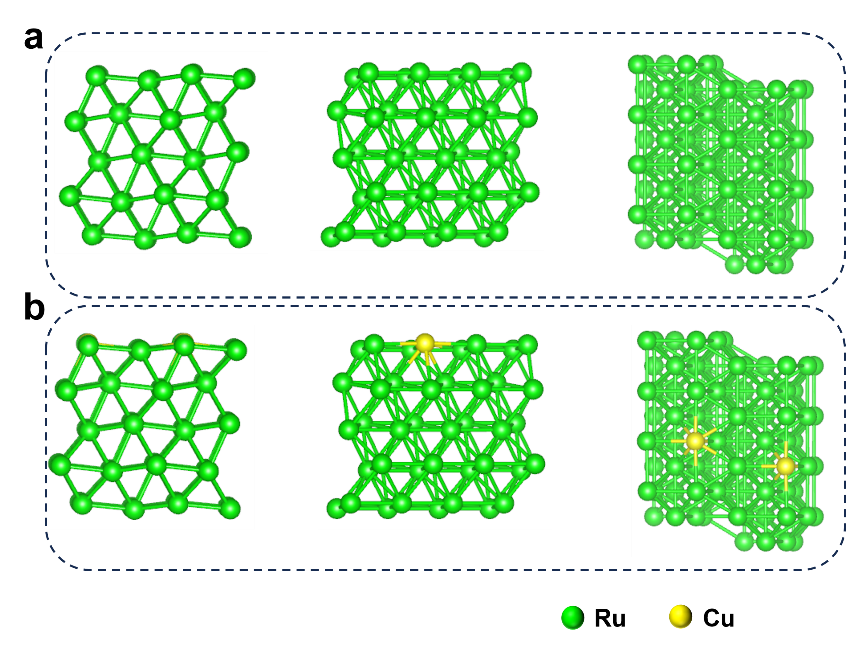


**Figure S19.** Theoretical models of (a) Ru (101) and (b) RuCu (101).


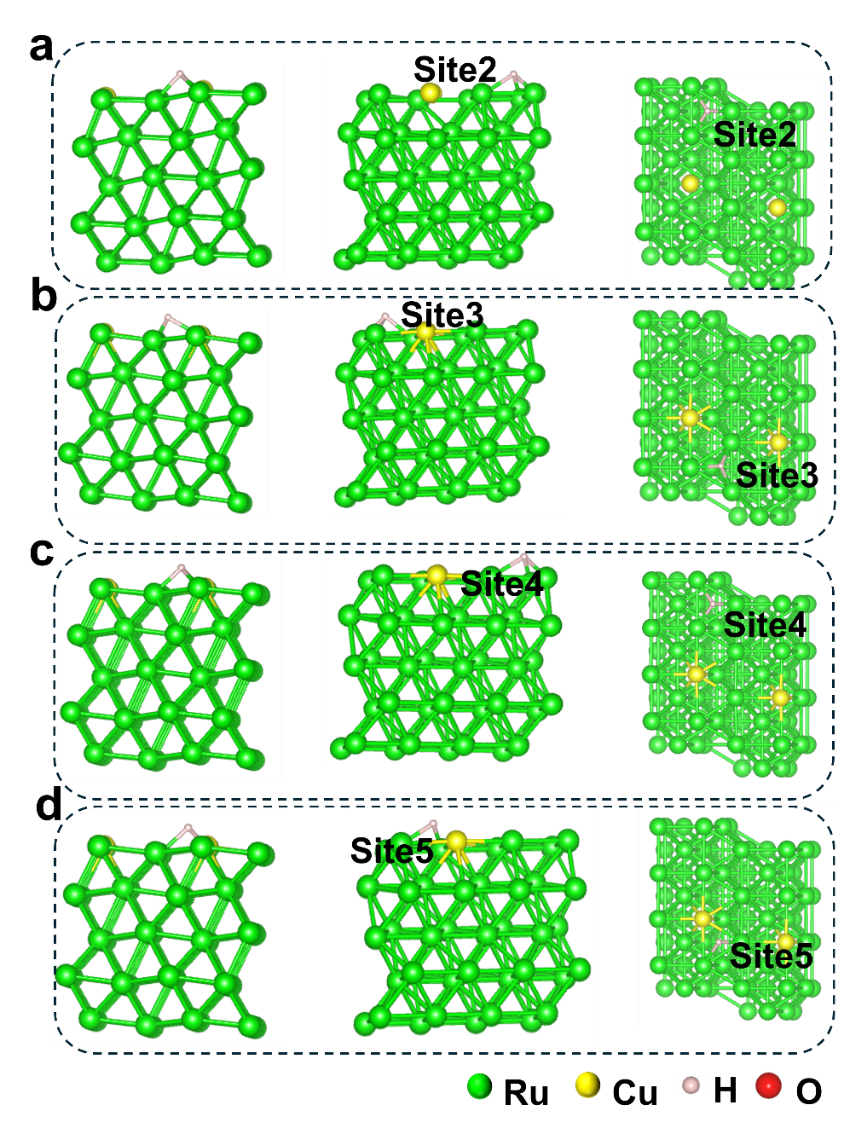


**Figure S20.** (a-d) Theoretical models of H adsorption on Site-(2-5) of RuCu (101).


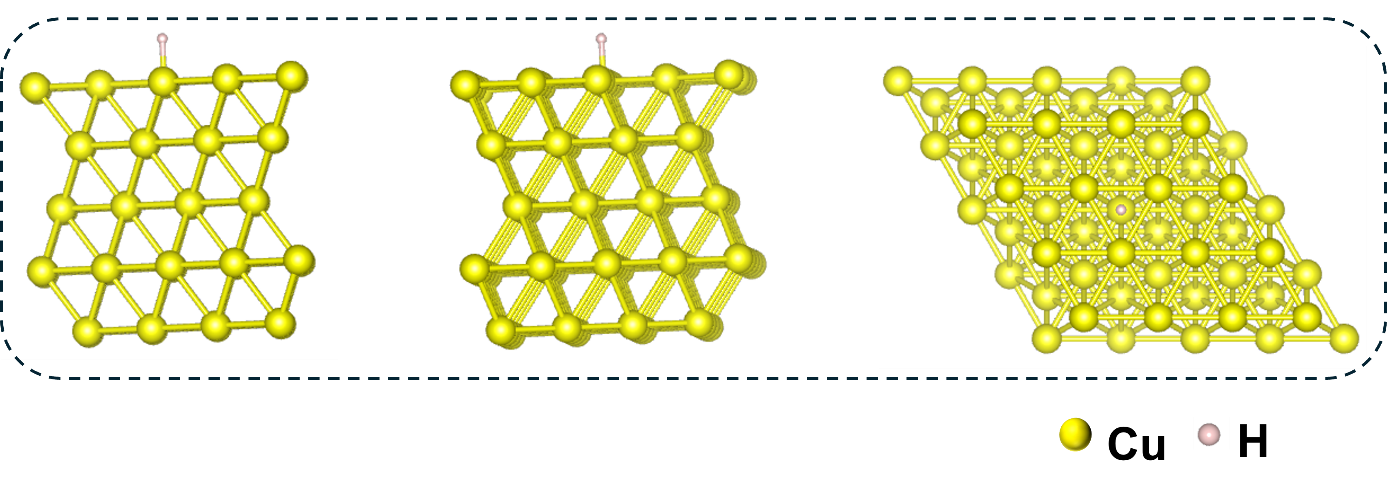


**Figure S21.** Theoretical models of H adsorption on Cu (111).


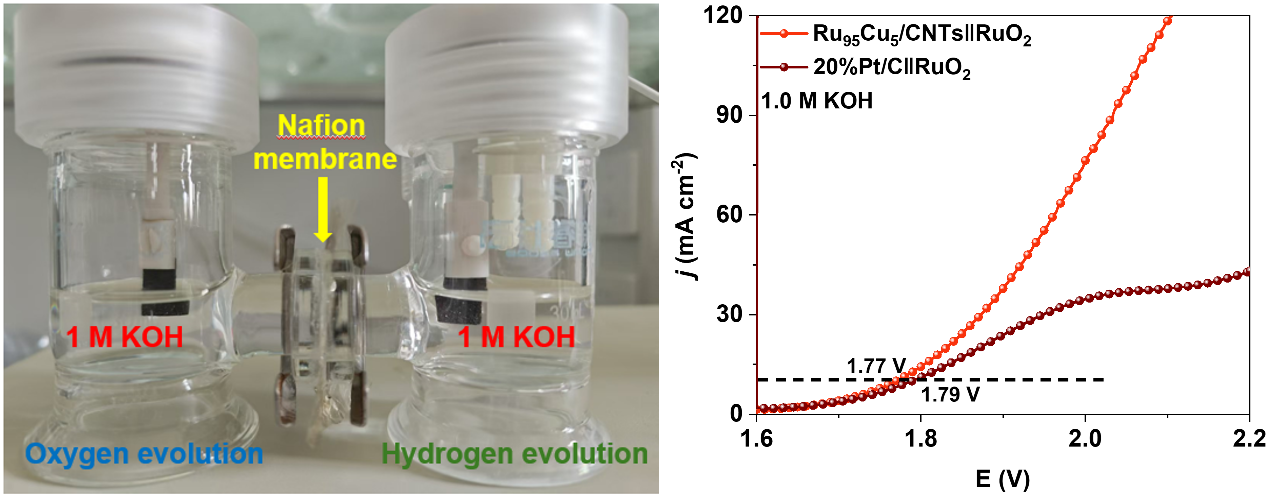


**Figure S22.** Optical image of experimental setup for overall water splitting and LSV curves of electrochemical overall water splitting performance of Ru_95_Cu_5_/CNTs||RuO_2_ catalysts and 20%Pt/C||RuO_2_ in 1.0 M KOH.
